# Efficacy and safety of direct oral anticoagulants for preventing venous thromboembolism in hospitalized cancer patients: a national multicenter retrospective cohort study

**DOI:** 10.3389/fphar.2024.1373635

**Published:** 2024-07-05

**Authors:** Shuyi Wu, Haiping Wang, Chunbao Li, Jingjing Tao, Xiaoli Zhu, Hengfen Dai, Hongfan Duan, Tian Hu, Miao Li, Fenfen Qu, Yun Wei, Chunhua Wang, Jinhua Zhang

**Affiliations:** ^1^ Department of Pharmacy, Fujian Maternity and Child Health Hospital College of Clinical Medicine for Obstetrics and Gynecology and Pediatrics, Fujian Medical University, Fuzhou, China; ^2^ Department of Pharmacy, The Second Hospital of Longyan Fujian Province, Longyan, China; ^3^ Department of Pharmacy, Jinjiang Municipal Hospital (Shanghai Sixth People’s Hospital Fujian Campus), Quanzhou, China; ^4^ Department of Pharmacy, BenQ Medical Center, The Affiliated BenQ Hospital of Nanjing Medical University, Nanjing, Jiangsu, China; ^5^ Department of Pharmacy, Red Cross Hospital of Yulin City, Yulin, China; ^6^ Department of Pharmacy, Affiliated Fuzhou First General Hospital of Fujian Medical University, Fuzhou, China; ^7^ Department of Pharmacy, The Second People’s Hospital of Baoshan City, Baoshan, China; ^8^ Department of Pharmacy, 3201 Hospital of Xi’an Jiaotong University Health Science Center, Hanzhong, Shaanxi, China; ^9^ Department of Pharmacy, The Second Hospital of Dalian Medical University Pharmacy Department Dalian, Dalian, China; ^10^ Department of Pharmacy, Yuncheng Central Hospital, Yuncheng, China; ^11^ Department of Pharmacy, Hunan Aerospace Hospital, Changsha, China; ^12^ Department of Neurosurgery, Fujian Medical University Union Hospital, Fuzhou, China

**Keywords:** direct oral anticoagulants, hospitalized cancer patients, venous thromboembolism, rivaroxaban, low-molecular-weight heparin

## Abstract

**Introduction:**

Studies on the use of direct oral anticoagulants (DOACs) for preventing venous thromboembolism (VTE) in hospitalized cancer patients are lacking. Therefore, we conducted a multicenter retrospective cohort study to evaluate the efficacy and safety of DOACs versus low-molecular-weight heparin (LMWH) for the primary prevention of VTE in hospitalized cancer patients.

**Methods:**

Clinical outcomes included thrombosis, VTE, other thrombosis, all bleeding, major bleeding, nonmajor bleeding, and all-cause death. A 1:1 cohort of rivaroxaban and LMWH patients was created by propensity score matching.

**Results:**

A total of 2,385 cancer patients were included in this study. During the 3-month follow-up period, 129 (5.4%) thrombosis events occurred, 63 (2.7%) of which were VTEs and 66 (2.8%) of which were other thrombosis events. All bleeding occurred in 163 (6.8%) patients, 68 (2.9%) had major bleeding, and 95 (4.0%) had nonmajor bleeding. All-cause deaths occurred in 113 (4.7%) patients. After adjusting for various confounders, the incidence of thrombosis and other thromboses was significantly lower in the rivaroxaban group than in the LMWH group [OR 0.543, 95% CI (0.343–0.859), *p* = 0.009; OR 0.461, 95% CI (0.241–0.883), *p* = 0.020]. There were no significant differences in incidence of VTE, total bleeding, major bleeding, nonmajor bleeding, or all-cause death.

**Conclusion:**

In oncology patients receiving thromboprophylaxis, rivaroxaban has a lower incidence of thrombosis and other thrombosis and a similar incidence of VTE as LMWH and does not increase the risk of bleeding. Rivaroxaban may be an attractive alternative to LMWH for preventing VTE in hospitalized cancer patients.

## 1 Introduction

Venous thromboembolism (VTE), which includes deep vein thrombosis (DVT) and pulmonary embolism (PE), is an important cause of morbidity and mortality in cancer patients (Donnellan and Khorana, 2017; Lyman et al., 2018a). Patients with cancer are more likely to experience VTE than noncancer patients are (Heit, 2015). Specific tumor populations at higher risk for VTE include patients with metastatic disease, multiple comorbidities, or infections; patients with a life expectancy <6 years; perioperative patients; and patients undergoing active treatment (Guntupalli et al., 2023). Novel therapies, including targeted therapies and immunosuppressive agents, have also been associated with an elevated risk of VTE, as have other chemotherapeutic agents (any systemic therapy appears to be a risk factor for VTE) (Lip et al., 2002).

Both the National Comprehensive Cancer Network (NCCN) and American Society of Medicine (ASCO) guidelines recommend low-molecular-weight heparin (LMWH) for thrombosis prophylaxis in hospitalized medical and surgical oncology patients (Streiff et al., 2022; Key et al., 2023). However, in patients requiring long-term anticoagulant medication, the discomfort of the injection site and the cost of LMWH are prominent issues (Yeh et al., 2014; Garcia et al., 2012; Kahn et al., 2012). Direct oral anticoagulants (DOACs), on the other hand, can be taken orally; they have fewer drug‒food interactions and generally do not need to be monitored, and their use prevents the discomfort of injections and frequent laboratory monitoring associated with the use of LMWH and vitamin K antagonists (Dong et al., 2020). In contrast, the easy application of DOACs provides a more convenient treatment option for cancer VTE patients with better medication adherence (Papakonstantinou et al., 2020).

However, there are fewer randomized controlled trials (RCTs) of DOACs for the primary prevention of VTE in cancer patients (Wang et al., 2019; Khorana et al., 2019; Becattini et al., 2022; Wang et al., 2022; Levine et al., 2012; Carrier et al., 2019a; Longo de Oliveira et al., 2022; Zhao et al., 2023; Guntupalli et al., 2020). Only four studies have compared the efficacy and safety of DOACs and LMWH (Wang et al., 2019; Longo de Oliveira et al., 2022; Zhao et al., 2023; Guntupalli et al., 2020). The CASSINI trial, one of the large studies evaluating the benefit of rivaroxaban for thromboprophylaxis in high-risk ambulatory cancer patients, did not show a significant reduction in the incidence of VTE or deaths due to VTE with rivaroxaban treatment during the trial period. However, during the intervention, rivaroxaban significantly reduced the incidence of VTE and was associated with a decreased incidence of major bleeding (Khorana et al., 2019). Based on this study, rivaroxaban has been routinely considered for primary prevention in high-risk cancer patients since 2019/2020 (Khorana score ≥2). For other DOACs, apixaban has been recommended by the NCCN guidelines as a prophylactic option for surgical oncology inpatients, especially for gynecological malignancies, as it was shown to be noninferior to LMWH in the study of Guntupalli et al. (Streiff et al., 2022; Guntupalli et al., 2020). In contrast, there are no relevant large RCTs on dabigatran or edoxaban for thromboprophylaxis in oncology patients; therefore, these drugs are not recommended for oncology thromboprophylaxis.

Tumor thromboprophylaxis is gaining increasing attention from clinicians worldwide. However, studies have focused more on the prophylactic use of LMWH, and studies on the use of DOACs for the prevention of VTE in hospitalized cancer patients are lacking. Therefore, we conducted a multicenter retrospective cohort study to evaluate the efficacy and safety of DOACs versus LMWH for the primary prevention of VTE in hospitalized cancer patients.

## 2 Methods

The methodology used in this study is broadly similar to that of our previously published study of the use of rivaroxaban vs. LMWH for preventing tumor thrombosis (Wu et al., 2023).

### 2.1 Study design

**FIGURE 1:**
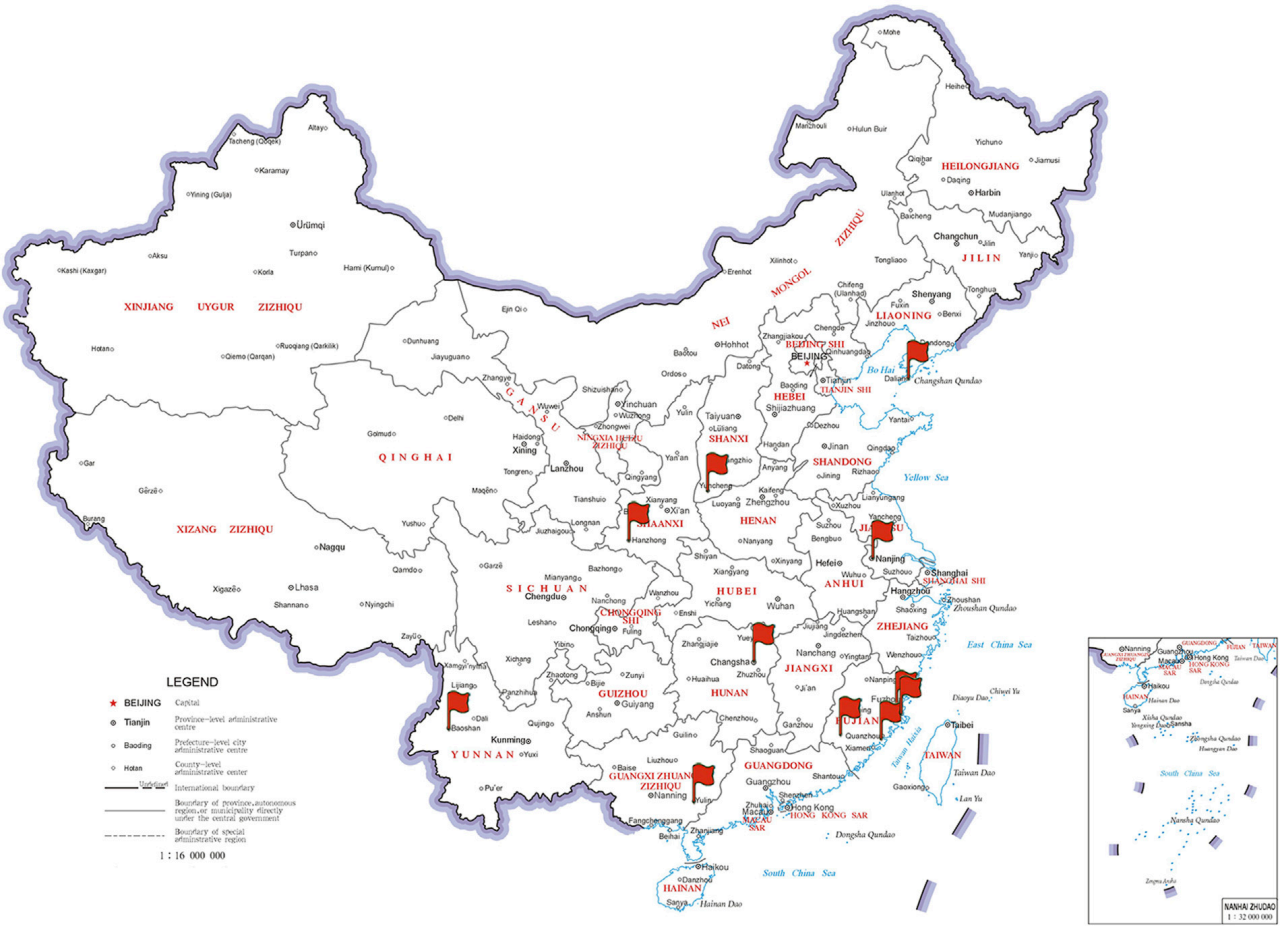
Subcenter distribution map.

This was a national multicenter retrospective cohort study. Inpatient medical and surgical cancer patients attending 12 hospitals in China from August 2016 to August 2023 were included (Supplementary Table S1). Figure 1 shows the multicenter distribution map. The Ethics Committee of Fujian Maternal and Child Health Hospital approved the protocol (study registration number: ChiCTR2300067734). Due to the retrospective nature of this study, the review committee waived the requirement for informed consent. The inclusion criteria for this study were as follows: (1) 18 years of age or older, (2) had a diagnosis of malignant solid tumor, and (3) used DOACs or LMWH for thromboprophylaxis. The exclusion criteria were as follows: (1) diagnosis of VTE at the time of enrollment; (2) use of LMWH and DOACs within the same regimen; (3) change to other oral anticoagulants during follow-up; (4) insufficient data for follow-up analysis; and (5) failure to undergo follow-up or less than 3 months of follow-up.

### 2.2 Data collection and definition

Demographic information was collected through the hospital information system, and the occurrence of clinical events was obtained through follow-up visits with patients or their relatives. We collected demographic and clinical information including (1) demographic characteristics [e.g., age, sex, body mass index (BMI)]; (2) lifestyle (e.g., smoking, drinking); (3) concomitant diseases (e.g., history of thromboembolic disease, hypertension, diabetes, congestive heart failure, coronary heart disease (CHD), hepatic abnormalities, renal abnormalities, and atherosclerosis); and (4) laboratory test parameters [e.g., white blood cell count (WBC), red blood cell count (RBC), hemoglobin (Hb), platelet count (PLT), albumin, creatinine, D-dimer, prothrombin time (PT), international standardized ratio (INR), activated partial thromboplastin time (APTT)]; (5) combination of drugs (e.g., antiplatelet drugs/NSAID, chemotherapeutic drugs, etc.); (6) history of surgery within a month, peripherally inserted central catheter (PICC), chemotherapy, etc. Drinking was defined as drinking >8 U/week; abnormal liver function was defined as chronic liver disease (e.g., cirrhosis) or biochemical evidence of significant hepatic dysfunction (e.g., bilirubin more than twice the upper limit of normal, aspartate aminotransferase/alanine aminotransferase/alkaline phosphatase more than three times the upper limit of normal); and abnormal renal function was defined as serum creatinine ≥200 mmol/L, chronic dialysis, or kidney transplantation (Lv et al., 2023). Information on thrombotic and hemorrhagic episodes and patient deaths after the administration of DOACs or LMWH was collected through follow-up. The follow-up period was 3 months. Finally, information and clinical events were collected from 2,685 patients, and the use of DOACs other than rivaroxaban was significantly lower (dabigatran in 5 patients, apixaban in 28 patients, and edoxaban in 2 patients). Therefore, this study focused on rivaroxaban and LMWH and did not analyze the other DOACs.

### 2.3 Outcomes

The primary outcome was VTE, and the secondary outcomes were thrombosis events, other thrombosis events, all bleeding, major bleeding, nonmajor bleeding, and all-cause death. VTE was defined as any symptomatic or incidentally detected proximal DVT of the lower or upper extremities, any nonfatal symptomatic or incidental PE, or death related to PE (Carrier et al., 2019b). Thrombosis events include VTE and other thrombosis (e.g., stroke, atrial thrombosis, catheter-related thrombosis, mesenteric thrombosis, heart attack, portal vein thrombosis, abdominal aortic wall thrombosis, and popliteal vein thrombosis). The International Society on Thrombosis and Hemostasis (ISTH) defines hemorrhage as bleeding leading to death occurring in a critical organ (intracranial, intraspinal, intraocular, retroperitoneal, intra-articular or pericardial, intramuscular versus fascial compartment syndrome) or associated with a decrease in hemoglobin level of at least 2 g/dL or transfusion of at least 2 units of red blood cells (Schulman et al., 2005). Nonmajor bleeding was defined as bleeding that did not meet the criteria for major bleeding as defined by the ISTH. All bleeding events included all bleeding, including major and nonmajor bleeding. All-cause deaths were defined as deaths from any cause.

### 2.4 Statistical analysis

Descriptive statistics for continuous variables are expressed as medians (interquartile ranges, IQRs), and descriptive variables are expressed as counts or percentages. Continuous variables were tested for normality, and t tests were used to compare the differences in continuous variables between the two groups of patients if they conformed to a normal distribution; nonparametric statistical tests were used if they did not conform to a normal distribution. The chi-square test was used to compare the distributions of categorical variables.

Logistic regression was used to analyze potential confounders affecting VTE, thrombosis, other thrombosis, all bleeding, major bleeding, nonmajor bleeding, and all-cause deaths. The covariates included age, sex, BMI, Khorana ≥2, tumor stage ≥ III, smoking status, drinking status, history of thrombosis, duration of anticoagulant use, hypertension, diabetes, heart failure, CHD, renal insufficiency, liver insufficiency, chronic obstructive pulmonary disease (COPD), atherosclerosis, history of surgery within a month, chemotherapy, alkylating agents, antimetabolites, antitumor antibiotics, topoisomerase inhibitors, botanical alkaloids, hormonal drugs, molecular targeted drugs, antiplatelet drugs/NSAIDs, WBC, RBC, PLT, PT, INR, APTT, D-dimer, albumin, and creatinine.

For further analysis, propensity score matching (PSM) was performed on the included covariates to create comparable rivaroxaban and LMWH cohorts in a 1:1 ratio—nearest-neighbor matching using a 0.02 caliper on the propensity score scale (Wang et al., 2014). Statistical analysis was performed using SPSS Statistics v. 22 (IBM Corporation, Armonk, NY, USA), and plotting was performed using R software (R [4.1.1], R Core Team [2021]). A two-sided *p*-value < 0.05 was considered to indicate statistical significance.

## 3 Results

### 3.1 Baseline characteristics

**FIGURE 2:**
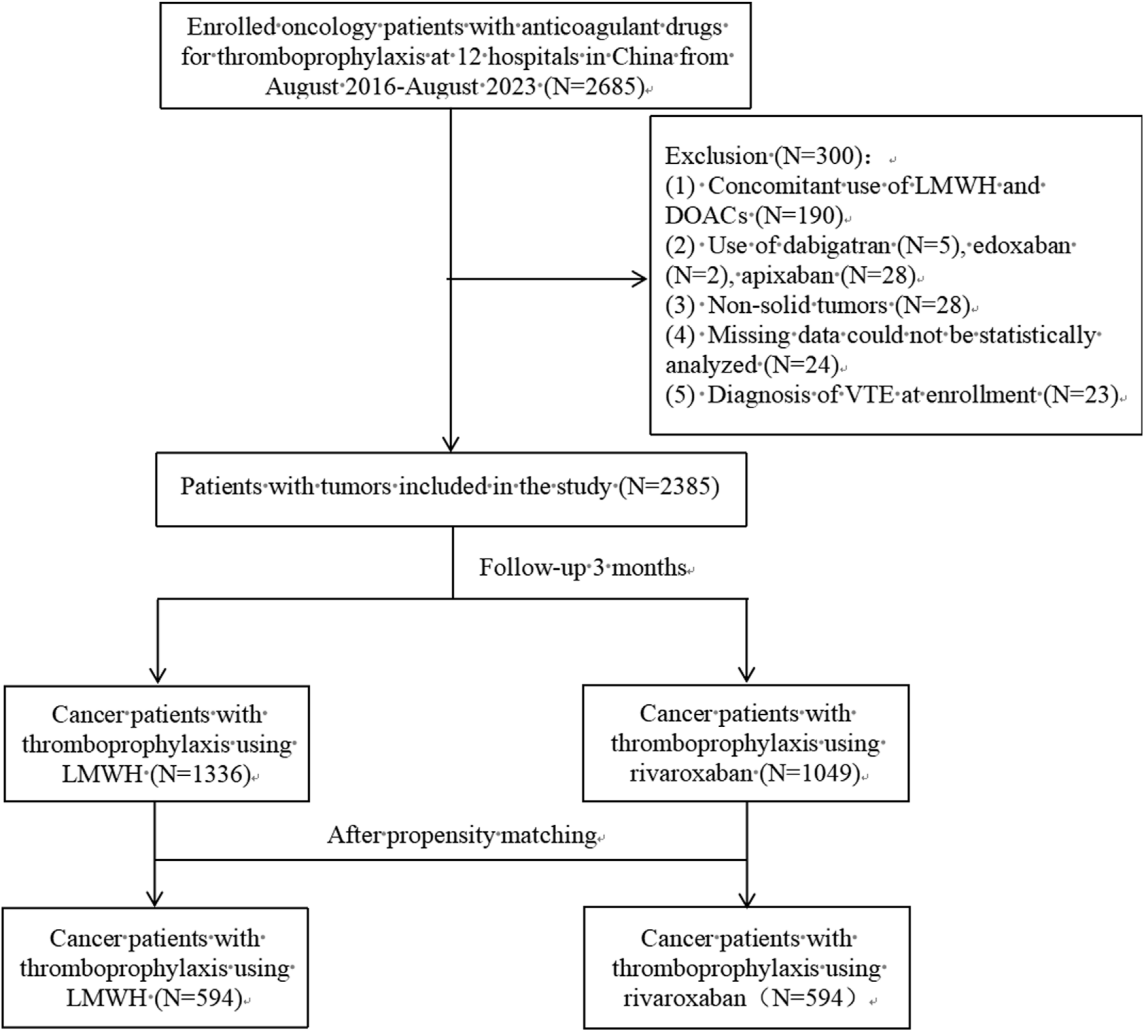
Study population selection.

A total of 2,385 oncology patients [1124 (47.1%) males, median age 62 years (IQR 52-70)] were enrolled in this study; 1333 patients used LMWH for thromboprophylaxis [595 (44.5%) males, median age 60 years (IQR 51-69)], and 1049 used rivaroxaban for thromboprophylaxis [529 (50.4%) males, median age 63 years (IQR 53-71)]. The inclusion flow chart is shown in Figure 2. Table 1 shows the baseline information of the patients. Lung cancer had the highest number of included patients, followed by cervical, breast, uterine, and ovarian cancers, as did the rivaroxaban and LMWH groups. The rate of pancreatic cancer was greater in the rivaroxaban group than in the LMWH group. The proportion of patients with cervical and uterine cancers was greater in the LMWH group. Moreover, no significant differences were found between the two groups for other tumor sites.

**TABLE 1:** Patient characteristics at baseline.

	All (N = 2,385)	LMWH (N = 1336)	Rivaroxaban (N = 1049)	*p*-Value
Sex/male, n (%)	1124 (47.1)	595 (44.5)	529 (50.4)	0.005
Age, median (IQR)	62 (52-70)	60 (51-69)	63 (53-71)	<0.001
BMI, median (IQR)	22.9 (20.5–25.1)	22.7 (20.4–24.9)	23.0 (20.6–25.3)	0.079
Khorana, n (%)				0.772
0	563 (23.6)	329 (24.6)	234 (22.3)	
1	991 (41.6)	556 (41.6)	435 (41.5)	
2	575 (24.1)	311 (23.3)	264 (25.2)	
3	203 (8.5)	110 (8.2)	93 (8.9)	
4	51 (2.1)	29 (2.2)	22 (2.1)	
5	2 (0.1)	1 (0.1)	1 (0.1)	
Khorana ≥2, n (%)	831 (34.8)	451 (33.8)	380 (36.2)	0.225
Tumor stage, n (%)				<0.001
I	661 (27.7)	407 (30.5)	254 (24.2)	
II	442 (18.5)	272 (20.4)	170 (16.2)	
III	605 (25.4)	336 (25.1)	269 (25.6)	
IV	677 (28.4)	321 (24.0)	356 (33.9)	
Tumor stage ≥ III, n (%)	1282 (53.7)	657 (49.2)	625 (59.6)	<0.001
Smoke,n(%)	293 (12.2)	180 (13.5)	113 (10.8)	0.046
Drink, n (%)	307 (12.8)	158 (11.8)	149 (14.2)	0.097
History of thrombosis,n (%)	324 (13.5)	103 (7.7)	221 (21.1)	<0.001
Anticoagulation time, median (IQR)	7 (4-14)	7 (3-10)	12 (7-30)	<0.001
Comorbidities, n (%)				
Hypertension	686 (28.7)	382 (28.6)	304 (29.0)	0.871
Coronary heart disease	110 (4.6)	58 (4.3)	52 (5.0)	0.540
Diabetes	342 (14.3)	179 (13.4)	163 (15.5)	0.155
Congestive heart failure	46 (1.9)	14 (1.0)	32 (3.1)	<0.001
COPD	51 (2.1)	25 (1.9)	26 (2.5)	0.382
Abnormal renal function	119 (4.9)	58 (4.3)	61 (5.8)	0.122
Abnormal liver function	236 (9.8)	132 (9.9)	104 (9.9)	0.978
Atherosclerosis	207 (8.6)	101 (7.6)	106 (10.1)	0.034
Surgery, n (%)^a^	960 (40.2)	672 (50.3)	288 (27.5)	<0.001
PICC,n (%)	850 (35.6)	472 (35.3)	378 (36.0)	0.754
Chemotherapy, n (%)	1055 (44.2)	521 (39.0)	534 (50.9)	<0.001
Tumor site, n (%)				
Lung	561 (23.5)	291 (21.8)	270 (25.7)	0.143
Cervix	261 (10.9)	163 (12.2)	98 (9.3)	0.026
Breast	227 (9.5)	137 (10.3)	90 (8.6)	0.167
Uterus	188 (7.9)	125 (9.4)	63 (6.0)	0.003
Ovaries	149 (6.2)	91 (6.8)	58 (5.5)	0.199
Esophagus	144 (6.0)	79 (5.9)	65 (6.2)	0.773
Rectum	138 (5.8)	77 (5.8)	61 (5.8)	0.956
Stomach	132 (5.5)	63 (4.7)	69 (6.6)	0.048
Colon	126 (5.3)	70 (5.2)	56 (5.3)	0.916
Liver	85 (3.6)	48 (3.6)	37 (3.5)	0.933
Pancreas	75 (3.1)	26 (1.9)	49 (4.7)	<0.001
Prostate	46 (1.9)	25 (1.9)	21 (2.0)	0.818
bladder	37 (1.6)	19 (1.4)	18 (1.7)	0.564
kidney	35 (1.5)	21 (1.6)	14 (1.3)	0.632
Bile ducts	20 (0.8)	7 (0.5)	13 (1.2)	0.057
Others^b^	161 (6.8)	94 (7.0)	67 (6.4)	0.531
Chemotherapeutic drug, n (%)				
Alkylating agents	462 (19.3)	187 (14.0)	275 (26.2)	<0.001
Antimetabolites	356 (14.9)	134 (10.0)	222 (21.2)	<0.001
Antitumor antibiotics	162 (6.7)	117 (8.8)	45 (4.3)	<0.001
Topoisomerase inhibitors	92 (3.8)	20 (1.5)	72 (6.9)	<0.001
Botanical alkaloid	289 (12.1)	118 (8.8)	171 (16.3)	<0.001
Hormone Drugs	513 (21.5)	280 (21.0)	233 (22.2)	0.491
Molecularly targeted drug	234 (9.8)	75 (5.6)	159 (15.2)	<0.001
Antiplatelet drug/NSAID, n (%)	404 (16.9)	245 (18.3)	159 (15.2)	0.045
Laboratory indicators, median (IQR)				
WBC	6.37 (4.94–8.67)	6.46 (5.09–8.90)	6.24 (4.66–8.41)	0.006
RBC	4.00 (3.46–4.48)	4.03 (3.50–4.50)	3.96 (3.40–4.44)	0.048
Hb	118 (102-133)	118 (101-133)	118 (102-133)	0.916
PLT	227 (173-195)	233 (181-305)	219 (164-285)	<0.001
PT	12.3 (11.3–13.3)	12.2 (11.2–13.2)	12.4 (11.4–13.5)	<0.001
INR	1.01 (0.94–1.11)	1.01 (0.93–1.09)	1.02 (0.95–1.14)	<0.001
APTT	31.4 (27.6–35.7)	31.4 (27.7–35.4)	31.6 (27.5–35.9)	0.672
D-Dimer	16.2 (14.9–17.3)	0.99 (0.39–2.93)	0.99 (0.44–2.93)	0.964
Albumin	37.9 (33.3–41.9)	37.7 (32.9–41.9)	38.3 (34.0–41.7)	0.141
Creatinine	67 (56-82)	66 (55-80)	68 (57-83)	0.045

LMWH: low-molecular-weight heparin; IQR: interquartile range; BMI: body mass index; COPD: chronic obstructive pulmonary disease; PICC: peripherally inserted central catheter; NSAID: nonsteroidal anti-inflammatory drug; WBC: white blood cell count; RBC: red blood cell count; Hb: hemoglobin; PLT: platelet count; PT: prothrombin time; INR: international standardized ratio; APTT: activated partial thromboplastin time.

^a^ A history of surgery within a month.

^b^ Other cancer sites include the nasopharynx, fallopian tubes, vagina, ureter, thyroid, tongue, and brain.

Regarding basic information, there were no significant differences between the rivaroxaban and LMWH groups regarding BMI, Khorana score, or alcohol consumption. A greater proportion of patients in the rivaroxaban group were male, were older, had a history of previous thrombosis, and had advanced tumors. A greater proportion of patients in the LMWH group smoked. At baseline, rivaroxaban was used longer than LMWH.

There were no significant differences in the presence of hypertension, diabetes, CHD, COPD, abnormal liver function, or abnormal renal function between the two groups of patients. In contrast, the rates of comorbid congestive heart failure and atherosclerosis were greater in the rivaroxaban group than in the LMWH group. In addition, a greater proportion of patients in the LMWH group underwent surgery within a month, while a greater proportion of patients in the rivaroxaban group underwent chemotherapy.

Regarding chemotherapeutic agents used in chemotherapy patients, the rivaroxaban group had a greater percentage of patients using alkylating agents, antimetabolites, topoisomerase inhibitors, botanical alkaloids, or molecularly targeted agents. In comparison, the LMWH group had a greater rate of use of antitumor antibiotics. Regarding laboratory indices, the median PT, median APTT, median INR, and median creatinine were greater in the rivaroxaban group than in the LMWH group. In contrast, the median WBC and median RBC counts were greater in the LMWH group.

### 3.2 Outcomes

During the 3-month follow-up period, 129 (5.4%) thrombosis events occurred, including 63 (2.7%) VTEs and 66 (2.8%) other thrombosis events. All bleeding occurred in 163 (6.8%) patients, 68 (2.9%) had major bleeding, and 95 (4.0%) had nonmajor bleeding. All-cause deaths occurred in 113 (4.7%) patients. The specific clinical events that occurred in the rivaroxaban and LMWH groups are shown in Table 2.

**TABLE 2:** Efficacy and safety outcomes.

Outcomes	All (N = 2,385)	LMWH (n = 1336)	Rivaroxaban (n = 1050)	Or (95% CI)	*p*-Value	AOR (95% CI)^a^	*p*-Value
Thrombosis, n (%)	129 (5.4)	76 (5.7)	53 (5.1)	0.882 (0.615–1.265)	0.495	0.543 (0.343–0.859)	0.009
VTE, n (%)	63 (2.7)	35 (2.6)	28 (2.7)	1.019 (0.616–1.687)	0.940	0.734 (0.393–1.370)	0.331
Other thrombosis, n (%)	66 (2.8)	41 (3.1)	25 (2.4)	0.771 (0.466–1.277)	0.311	0.461 (0.241–0.883)	0.020
All bleeding, n (%)	163 (6.8)	79 (5.9)	84 (8.0)	1.385 (1.007–1.904)	0.044	1.233 (0.855–1.777)	0.262
Major bleeding, n (%)	68 (2.9)	28 (2.1)	40 (3.8)	1.852 (1.135–3.023)	0.012	1.573 (0.891–2.778)	0.119
Nonmajor bleeding, n (%)	95 (4.0)	51 (3.8)	44 (4.2)	1.103 (0.731–1.665)	0.640	1.090 (0.677–1.753)	0.724
All-caused deaths, n (%)	113 (4.7)	54 (4.0)	59 (5.6)	1.415 (0.969–2.065)	0.071	1.465 (0.923–2.326)	0.105

LMWH: low-molecular-weight heparin; 95% CI: confidence interval; OR: odds ratio; AOR: adjusted odds ratio; VTE: venous thromboembolism.

^a^ Adjust for age, sex, BMI, Khorana ≥2, tumor stage ≥ III, smoking, drinking, history of thrombosis, anticoagulation time, hypertension, diabetes, heart failure, congestive heart failure, abnormal renal function, abnormal liver function, COPD, atherosclerosis, history of surgery in the last month, chemotherapy, alkylating agents, antimetabolites, antitumor antibiotics, topoisomerase inhibitors, botanical alkaloid, hormonal drugs, molecularly targeted drugs, antiplatelet agents/NSAID, WBC, RBC, hb, PLT, PT, INR, APTT, D-Dimer, albumin, creatinine.

#### 3.2.1 Thrombosis events

Thrombosis occurred in 53 (5.1%) patients in the rivaroxaban group; 28 (4.0%) had VTEs, and 25 (2.4%) had other thromboses. Seventy-six (5.7%) patients in the LMWH group experienced thrombosis, 35 (2.6%) of whom had VTEs and 41 (3.1%) had other thromboses.

The thrombosis rate was greater in the LMWH group than in the rivaroxaban group, but there was no significant difference between the two groups [OR = 0.882, 95% CI = 0.615–1.265; *p* = 0.495]. After adjusting for various confounders, the thrombosis rate was significantly greater in the LMWH group than in the rivaroxaban group [OR = 0.543, 95% CI = 0.343–0.859; *p* = 0.009]. The effects of anticoagulants and potential risk factors for thrombosis in oncology patients are shown in Supplementary Figure S1. The combined use of antitumor antibiotics, hormonal drugs, CHD, and PICC is a risk factor for thrombosis in oncology patients.

The incidence of VTE was greater in the LMWH group than in the rivaroxaban group, but there was no significant difference between the two groups [OR 1.019, 95% CI (0.616–1.687), *p* = 0.940]. After adjusting for confounders, there was still no significant difference in VTE events between the LMWH and rivaroxaban groups [OR 0.817, 95% CI (0.444–1.505), *p* = 0.517]. The effects of anticoagulants and potential risk factors for VTE in oncology patients are shown in Supplementary Figure S2. Comorbid CHD, COPD, PICC, and the use of hormonal drugs are risk factors for VTE in oncology patients.

The incidence of other thromboses was greater in the LMWH group than in the rivaroxaban group, but there was no significant difference between the two groups [OR = 0.771, 95% CI = 0.466–1.277; *p* = 0.311]. After adjusting for various confounders, the incidence of thrombosis in other patients was greater in the LMWH group than in the rivaroxaban group [OR = 0.461, 95% CI = 0.241–0.883; *p* = 0.020]. The effects of anticoagulants and potential risk factors for thrombosis in oncology patients are shown in Supplementary Figure S3. Hypertension, CHD, atherosclerosis, PICC, and the use of antitumor antibiotics are risk factors for other thrombosis in oncology patients.

#### 3.2.2 Bleeding events

Bleeding events occurred in 84 (8.0%) patients in the rivaroxaban group, 40 (3.8%) of whom had major bleeding and 44 (4.2%) of whom had nonmajor bleeding. Bleeding events occurred in 79 (5.9%) patients in the LMWH group, 28 (2.1%) of whom had major bleeding and 51 (3.8%) of whom had nonmajor bleeding.

The incidence of all bleeding events was significantly greater in the rivaroxaban group than in the LMWH group [OR 1.385, 95% CI (1.007–1.904), *p* = 0.004]. After adjusting for various confounders, the difference between the two groups was not significant [OR 1.233, 95% CI (0.855–1.777), *p* = 0.262]. The effects of anticoagulants and potential risk factors for all bleeding in tumor patients are shown in Supplementary Figure S4. Abnormal renal function, elevated WBC counts, and increased APTT were risk factors for all bleeding in tumor patients.

The incidence of major bleeding was significantly greater in the rivaroxaban group than in the LMWH group [OR 1.852, 95% CI (1.135–3.023), *p* = 0.012]. After adjusting for various confounders, we detected no significant difference in major bleeding events between the rivaroxaban and LMWH groups [OR 1.573, 95% CI (0.891–2.778), *p* = 0.119]. The effects of anticoagulants and potential risk factors for major bleeding in oncology patients are shown in Supplementary Figure S5. Increased APTT, abnormal renal function, abnormal liver function, and tumor stage > III are risk factors for major bleeding in tumor patients.

The incidence of nonmajor bleeding was greater in the rivaroxaban group than in the LMWH group, but the difference was not statistically significant [OR 1.103, 95% CI (0.731–1.665), *p* = 0.640]. After adjusting for various confounders, there was still no significant difference between the rivaroxaban and LMWH groups regarding nonmajor bleeding [OR 1.090, 95% CI (0.677–1.753), *p* = 0.724]. The effects of anticoagulants and potential risk factors for nonmajor bleeding in oncology patients are shown in Supplementary Figure S6. Increased APTT is a risk factor for nonmajor bleeding in oncology patients.

#### 3.2.3 All-cause deaths

During the 3-month follow-up period, 113 patients died. Of these, 59 (5.6%) were in the rivaroxaban group, and 54 (4.0%) were in the LMWH group. There was no significant difference in all-cause deaths between the rivaroxaban and LMWH groups [OR 1.415, 95% CI (0.969–2.065), *p* = 0.071]. After adjusting for various confounders, there was still no significant difference in all-cause deaths between the two groups [OR 1.465, 95% CI (0.923–2.326), *p* = 0.105]. The effects of anticoagulants and potential risk factors on all-cause deaths in patients with tumors are shown in Supplementary Figure S7. Male sex, increasing age, tumor stage ≥ III, PICC, and elevated WBC count are risk factors for all-cause death in tumor patients.

### 3.3 PSM cohort

**FIGURE 3:**
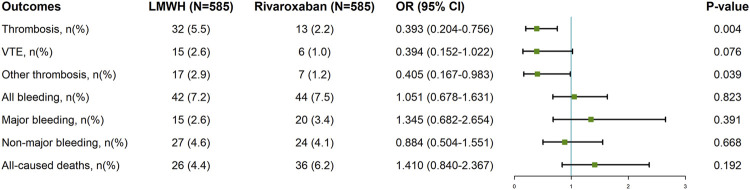
Clinical Outcomes of Rivaroxaban and LMWH in tumor patients after propensity score matching. LMWH: low molecular weight heparin; 95% CI: confidence interval; OR: odds ratio; VTE: venous thromboembolism.

We used PSM to identify 585 patients in each group with comparable baseline characteristics (Supplementary Table S2). In the PSM cohort, the rivaroxaban group had a lower incidence of thrombosis and other thromboses than did the LMWH group [OR = 0.393, 95% CI = 0.204–0.756; *p* = 0.004; OR = 0.405, 95% CI = 0.167–0.983; *p* = 0.039]. There was no significant difference between the two groups regarding VTE incidence, total bleeding, major bleeding, nonmajor bleeding, or all-cause deaths (Figure 3).

### 3.4 Subgroup analysis

We performed subgroup analyses of a larger number of patients with lung cancer. Supplementary Table S3 shows baseline information after PSM in patients with lung cancer. Supplementary Table S4 shows the clinical outcomes of patients with lung cancer who received anticoagulants for thrombosis prophylaxis. Among 242 lung cancer patients, 13 had thromboses, including 6 with VTEs and 7 with other thromboses. Seventeen bleeding events occurred, including 9 major bleeding events and 8 minor bleeding events, and 21 all-cause deaths occurred. All bleeding was significantly greater in the rivaroxaban group than in the LMWH group [OR 3.521, 95% CI (1.114–11.128), *p* = 0.024]. There were no significant differences between the two groups in terms of thrombosis, VTE, other thrombosis, major bleeding, nonmajor bleeding, or all-cause deaths.

We also performed subgroup analyses of oncology patients who underwent surgery within a month. Supplementary Table S5 shows the baseline information of oncology surgery patients after PSM. Supplementary Table S6 shows the clinical outcomes of anticoagulant use in oncology surgery patients. Among the 336 surgical patients, the all-cause death rate was significantly greater in the rivaroxaban group than in the LMWH group [*p* = 0.030]. In contrast, there were no significant differences between rivaroxaban and LMWH regarding thrombosis, VTE, other thrombosis, all bleeding, major bleeding, or nonmajor bleeding.

In addition, we performed subgroup analyses of chemotherapy patients in the rivaroxaban group, the LMWH group and patients with a documented number of chemotherapy times. Supplementary Table S7 presents baseline information on oncological chemotherapy patients after PSM. Among the 432 chemotherapy patients, the incidence of thrombosis and other thrombosis was greater in the LMWH group than in the rivaroxaban group [OR 0.399, 95% CI (0.171–0.932), *p* = 0.029; OR 0.239, 95% CI (0.067–0.861), *p* = 0.018]. There were no significant differences between LMWH and rivaroxaban in terms of the incidence of VTE, all bleeding, major bleeding, nonmajor bleeding, or all-cause deaths (Supplementary Table S8). Patients in the LMWH and rivaroxaban groups were divided into 4 groups according to the duration of chemotherapy: 1-2, 3-4, 5-6, and ≥7 times of chemotherapy. Supplementary Table S9 and Supplementary Table S10 show the number of chemotherapy times and thrombosis events for patients treated with chemotherapy in the rivaroxaban and LMWH groups, respectively. The highest number of patients in both groups were treated with chemotherapy 1-2 times. The highest rate of thrombosis was observed in patients treated with chemotherapy 3-4 times in the rivaroxaban group (18.5%) and chemotherapy 1-2 times in the LMWH group (9.8%). The thrombosis rate was significantly greater in patients who received chemotherapy 3-4 than in those who received chemotherapy 1-2. In contrast, the thrombosis rate decreased but was not significantly different in the LMWH group for patients who underwent 3-4, 5-6, or ≥7 timesof chemotherapy (*p* = 0.271; *p* = 0.093; *p* = 0.053; *p* = 0.142).

## 4 Discussion

Our multicenter retrospective cohort study included 12 hospitals in East, Central, South, North, Northeast, Southwest, and Northwest China. The results of this study provide interesting preliminary evidence to support the use of rivaroxaban for thromboprophylaxis in cancer patients who are unable or unwilling to use injectable LMWH. Our study had the following findings: (1) Thromboprophylaxis with rivaroxaban had fewer thrombosis and other thrombotic incidences than LMWH over a 3-month follow-up period. There were no significant differences in VTE incidence, all bleeding, major bleeding, nonmajor bleeding, or all-cause deaths between patients treated with rivaroxaban and those treated with LMWH. (2) In patients with lung cancer, there was a greater risk of all bleeding with rivaroxaban than with LMWH, but there were no significant differences in thrombosis, VTE, other thromboses, major bleeding, nonmajor bleeding, or all-cause death. (3) Thromboprophylaxis with LMWH was used more often in patients with tumors who underwent surgery within a month. Surgical oncology patients who used rivaroxaban for thromboprophylaxis had a greater all-cause death rate than did those who used LMWH. (4) Thromboprophylaxis with rivaroxaban is used more often in chemotherapy patients.

There are fewer real-world studies on the use of rivaroxaban vs. LMWH for preventing tumor thrombosis. Our study revealed significant differences between rivaroxaban and LMWH for thrombosis and other thrombosis, with rivaroxaban having a lower incidence. LMWH accelerates the inhibitory effect of antithrombin on activated factor X during the conversion of prothrombin to thrombin (Hirsh et al., 2008). Rivaroxaban is a factor Xa inhibitor that directly and reversibly binds and competitively inhibits factor Xa. It is 10,000-fold more selective for factor Xa than for other factors and does not require cofactors to exert its anticoagulant effect (Eriksson et al., 2011). It seems plausible that, compared with LMWH, rivaroxaban is associated with enhanced antithrombotic effects, which act indirectly on factor X (Faqah et al., 2020). However, in our previously published retrospective cohort study with a small sample size, no significant differences in thrombosis or bleeding events between the rivaroxaban and LMWH groups were observed (Wu et al., 2023). We found that the incidence of thrombosis in the present study was lower than that in all previous studies. These differences may be because LMWH needs to be injected for use. If patients are injected themselves, there may be injection discomfort and infection at the injection site, which reduces patient medication compliance. Therefore, most hospitals in China do not use LMWH as a medication and are discharged from the hospital, which leads to insufficient thromboprophylaxis in some patients and thus an increased incidence of thrombosis. Previous studies analyzed only 600 patients from 2 hospitals, whereas the problem of inadequate thromboprophylaxis combined with LMWH use in hospitalized patients is now more pronounced in larger sample sizes. Therefore, patients who use LMWH during hospitalization should be evaluated for thrombosis risk at discharge, and a decision should be made whether to continue other anticoagulants (e.g., rivaroxaban) after discharge.

The VALERIA trial was a randomized controlled study conducted by Oliveira et al. to evaluate the preventive effect of rivaroxaban versus enoxaparin on postoperative VTE in patients with gynecologic pelvic cancer (Longo de Oliveira et al., 2022). The study revealed no significant difference between rivaroxaban and LMWH in preventing postoperative VTE or bleeding (either major or minor) in gynecologic oncology patients. This finding is in partial agreement with our study. However, significant differences were observed in our study between thrombosis and other thromboses. The incidence of thrombosis in our study was greater than that in Oliveira et al. These differences may be due to the large number of malignant tumor categories included in the present study. Major cancer sites at risk of VTE, such as pancreatic, renal, gastric, ovarian, lung, and esophageal cancer sites, were included in this study (Wun and White, 2009; Lyman et al., 2018b; Chew et al., 2006). Oliveira et al. included only postoperative gynecologic oncology patients. The risk of VTE varies by cancer site, and patients with the highest incidences of VTE are usually diagnosed with gastric cancer, pancreatic cancer, or primary brain tumors (Moik et al., 2020). In contrast, patients with breast cancer and prostate cancer have a lower risk of VTE (Moik et al., 2020; Mulder et al., 2021; Grilz et al., 2021). In addition, the duration of thrombosis prevention by anticoagulants differed. Oliveira et al. used rivaroxaban and LMWH for 30 days, whereas in our study, the median duration of prevention was 12 (IQR 7-30) for rivaroxaban and 12 (IQR 7-30) for LMWH. All of these factors may have contributed to the discrepancy between the results of the present study and those of Oliveira et al.

We analyzed the risk factors for thrombosis, bleeding, and all-cause death in hospitalized oncology patients. CHD and PICC were risk factors for thrombosis. Hypertension and atherosclerosis are risk factors for other types of thrombosis. CHD patients are prone to thrombosis due to increased blood viscosity, vascular endothelial damage, and other factors. PICCs aggravate the hypercoagulable state of blood in tumor patients and increase the risk of thrombosis. The use of hormonal drugs increases the risk of thrombosis and VTE in oncology patients, but the underlying mechanisms are not fully understood (Abou-Ismail et al., 2020). It has been shown that hormonal drugs increase thrombin production and D-dimer levels and thrombin generation (Kluft and Lansink, 1997; Godsland et al., 2000). In addition, hormone therapy plays a role in regulating endothelial function. Several reports have shown that estrogen has a dose-dependent effect on the expression of matrix metalloproteinases, which disrupt collagen and elastin in the endothelium, thereby leading to venous stasis and increasing vascular permeability and promoting VTE formation (Fawer et al., 1978). Antitumor antibiotics are also risk factors for thrombosis in cancer patients. Antitumor antibiotics can impair the production of NO, endothelin-1, neuromodulin, thrombomodulin, and thromboxane B2 by endothelial cells, leading to vasoconstriction and thrombosis (Podyacheva et al., 2023). Cancer stage is significantly associated with the risk of venous thromboembolism (Elalamy et al., 2023). However, in this study, a correlation between tumor stage and thrombosis was not found; rather, cancer stage was found to increase the risk of bleeding and all-cause death. Abnormal renal function, elevated WBC counts, and elevated APTT increased the risk of bleeding. Abnormal renal function is associated with increased inflammatory and procoagulant biomarkers, including C-reactive protein, fibrinogen, factor VII, and factor VIII (Shlipak et al., 2003; Shah et al., 2013). Additionally, abnormal renal function was included in the HAS-BLEED (Pisters et al., 2010) score, which suggests that it is associated with an increased risk of bleeding. A potential mechanism by which leukocytosis may indirectly contribute to bleeding events is that increased WBC concentrations may reduce platelet-mediated procoagulation through interactions with leukocytes, thereby affecting whole-blood procoagulant activity (Perussia et al., 1982). Zhou et al. also observed a J-shaped correlation between WBC and bleeding in a real-world study of patients with NVAF, with elevated WBC associated with an increased risk of bleeding to the right of the inflection point (6.75 × 109/L) (Zhou et al., 2019). Therefore, it is worth further investigating whether the WBC count is linearly correlated with bleeding risk in cancer patients.

We performed a subgroup analysis of lung cancer patients. We found no significant difference between rivaroxaban and LMWH in preventing thrombosis, but the incidence of all bleeding was greater in the rivaroxaban group than in the LMWH group. This result may be related to the greater coagulation perturbation caused by rivaroxaban (Fawer et al., 1978). The findings of the lung cancer subgroup differed from those of Zhao et al.'s study of postoperative thromboprophylaxis in lung cancer patients (Zhao et al., 2023). Zhao et al. conducted a randomized, nonefficacy trial comparing rivaroxaban versus nadroparin for thromboprophylaxis after thoracic surgery for lung cancer. The results showed that rivaroxaban did not increase the risk of bleeding compared to LMWH. A possible reason for these different results is that Zhao et al. included patients with postoperative lung cancer. In contrast, in our subgroup analysis, approximately 22% of the lung cancer patients underwent surgery, and almost 45% underwent chemotherapy. For lung cancer patients, clinical outcomes may be influenced by chemotherapy and surgery. Unfortunately, we could not analyze surgical lung cancer patients or chemotherapy lung cancer patients separately due to sample size limitations.

Our study showed that the proportion of patients with a history of surgery within 1 month was greater in the LMWH group than in the rivaroxaban group. In contrast, more patients in the rivaroxaban group were treated with chemotherapy, which may be an indication of practice preference. These differences may be because, for patients requiring surgery, physicians preferred LMWH for thromboprophylaxis based on the recommendations of the NCCN and ASCO guidelines. In contrast, in patients treated with chemotherapy, physicians preferred to discharge patients on rivaroxaban to prevent thrombosis. A subgroup analysis of patients with tumors treated with surgery revealed that rivaroxaban was noninferior to LMWH in preventing thrombosis and did not increase the risk of bleeding. Nevertheless, all-cause deaths were greater in the rivaroxaban group. These results may suggest that although the advantages of rivaroxaban in thromboprophylaxis can be considered, LMWH may be a more appropriate choice for thromboprophylaxis in oncology patients undergoing surgery in terms of survival.

Chemotherapy is a risk factor for thrombosis in patients with tumors (Sevestre and Soudet, 2020). Our subgroup analysis of hospitalized oncology patients treated with chemotherapy and duration of chemotherapy showed that among patients treated with chemotherapy, oncology patients in the rivaroxaban group had a lower incidence of thrombosis and other thrombosis than did those in the LMWH group and did not have an increased risk of bleeding or all-cause death. These findings may suggest that rivaroxaban may be more advantageous for thromboprophylaxis in chemotherapy patients. Based on the results of this study and the fact that rivaroxaban is used orally without monitoring and has better medication compliance, rivaroxaban may be a more appropriate choice for chemotherapy patients with thromboprophylaxis. The risk of thrombosis may vary with the duration of chemotherapy. The risk of thrombosis was greater in the rivaroxaban group for patients treated with 3-4 chemotherapy times than for patients treated with 1-2 chemotherapy times, whereas no significant difference was found for subsequent increases in the number of chemotherapy times compared with the number of chemotherapy times (1-2). In the LMWH group, no corresponding increase in thrombotic risk was observed with increasing numbers of chemotherapy treatments, with the highest risk of thrombosis occurring in patients treated with chemotherapy 1-2 times. A possible explanation is that after receiving one chemotherapy treatment at hospital A, the patients did not receive another chemotherapy or were transferred to hospital B for treatment, which was not queried in the medical records system of hospital A. The number of patients in the LMWH group decreased with the number of chemotherapy times. These findings may also explain the decrease in the number of patients in the LMWH group as the number of chemotherapy sessions increased.

The NCCN guidelines recommend that inpatient medical oncology patients received prophylaxis. According to the NCCN guidelines, patients receiving prophylaxis should persist for the duration of the hospital stay, 6–14 days, or until the patient is fully ambulatory. For inpatient surgical patients, the recommendation is to provide prevention for 7–10 days or until the patient is fully ambulatory (Streiff et al., 2022). However, the continued use of parenteral anticoagulants to prevent cancer-related thrombosis is challenging (Schaefer et al., 2021). Cost, adhesion, pain, and bruising at the injection site are also problems (Gómez-Outes et al., 2014). Our findings suggest that oral rivaroxaban may be an alternative to parenteral LMWH in cancer patients. Overall, compared with LMWH, rivaroxaban had a lower incidence of thrombosis and did not increase the incidence of bleeding during the 3-month follow-up period. Combined with the results of subgroup analysis, these findings indicate that LMWH may be a more appropriate choice for thromboprophylaxis in patients with surgically treated tumors. In contrast, rivaroxaban may be a more appropriate choice for patients with chemotherapeutic tumors. However, the risk of thrombosis differs for different cancer sites, and the choice of anticoagulant for thromboprophylaxis in different cancer sites deserves further analysis. The number of comorbidities also leads to an increased risk of VTE in hospitalized cancer patients (Mulder et al., 2021), and all categories of antitumor therapy (chemotherapy, targeted therapy, hormonal therapy, immunotherapy) are associated with a significant increase in the risk of VTE (Abou-Ismail et al., 2020). Therefore, additional attention should be given to thromboprophylaxis in cancer patients with comorbidities, especially those with cardiovascular diseases (coronary artery disease, atherosclerosis, etc.), as well as the use of chemotherapeutic agents or hormonal, immune, and targeted therapies. Patients with tumors and abnormal liver and kidney function need to be aware of the occurrence of bleeding when using anticoagulant drugs.

This study is the first multicenter retrospective study in China to compare the efficacy and safety of rivaroxaban and LMWH for preventing tumor thrombosis, providing a reference solution for selecting thromboprophylaxis for real-world tumor patients. At the same time, there are several limitations to this study. First, due to the retrospective nature of this study, there may be incomplete information about the results. Elderly patients constitute the majority of patients, and it is difficult to avoid the occurrence of unclear or confusing memories during follow-up. Due to the large sample size, a large number of patients were lost to follow-up. However, this did not significantly impact the analysis of outcomes, as most patients could be followed up through the medical records system and telephone follow-up. Besides, length of hospitalization affects the choice of thromboprophylaxis; however, this study failed to collect the length of hospitalization of patients. Second, other DOACs could not be analyzed due to the small sample size of patients receiving other DOACs. Finally, this study analyzed only subgroups of lung cancer patients and failed to analyze other cancers associated with high VTE risk (e.g., gastric cancer) due to the limited sample size. In future studies, additional samples could be collected to analyze the effects of different cancer sites and treatments (e.g., surgery, chemotherapy, targeted therapy, immunotherapy, etc.) on tumor thromboprophylaxis.

## 5 Conclusion

In thromboprophylaxis for oncology patients, rivaroxaban has a lower incidence of thrombosis and other thrombosis, a similar incidence of VTE as LMWH, and does not increase the risk of bleeding. Rivaroxaban may be an attractive alternative to LMWH for preventing VTE in cancer patients. Larger and more robust prospective studies are certainly needed to confirm our findings.

## Data Availability

The original contributions presented in the study are included in the article/Supplementary Material, further inquiries can be directed to the corresponding author.
