# Supplementary material for: Efficacy and safety of direct oral anticoagulants for preventing venous thromboembolism in hospitalized cancer patients: a national multicenter retrospective cohort study
Source: Front Pharmacol. 2024 Jul 5;15:1373635. doi: 10.3389/fphar.2024.1373635 (PMC11257898; doi:10.3389/fphar.2024.1373635)
Supplement: Supplementary file 1 [file DataSheet1.docx]

Supplementary Material

# Supplementary Tables

**Table 1** List of 12 multi-center hospitals

| **Number** | **Hospital** | **Investigators** |
| --- | --- | --- |
| 1 | Fujian Medical University Union Hospital | Chunhua Wang |
| 2 | The Second Hospital of Longyan Fujian Province | Haiping Wang |
| 3 | Jinjiang Municipal Hospital (Shanghai Sixth People's Hospital Fujian Campus) | Chunbao Li |
| 4 | BenQ Medical Center,The Affiliated BenQ Hospital of Nanjing Medical University | Jingjing Tao |
| 5 | Red Cross Hospital of Yulin City | Xiaoli Zhu |
| 6 | Affiliated Fuzhou First General Hospital of Fujian Medical University | Hengfen Dai |
| 7 | The Second People's Hospital of Baoshan City | Hongfan Duan |
| 8 | 3201 Hospital of Xi’an Jiaotong University Health Science Center | Tian Hu |
| 9 | The second hospital of Dalian Medical University Pharmacy Dept. | Miao Li |
| 10 | Yuncheng Central Hospital | Fenfen Qu |
| 11 | Hunan Aerospace Hospital | Yun Wei |
| 12 | Fujian Maternity and Child Health Hospital College of Clinical Medicine for Obstetrics & Gynecology and Pediatrics, Fujian Medical University | Jinhua Zhang |

**Table 2** Baseline characteristics after propensity score matching

|  | **LMWH (N=585)** | **Rivaroxaban (N=585)** | **P-value** |
| --- | --- | --- | --- |
| Sex/male, n(%) | 317 (54.2) | 302 (51.6) | 0.380 |
| Age, median (IQR) | 64 (54-71) | 62 (53-70) | 0.119 |
| BMI, median (IQR) | 22.8 (20.5-25.0) | 22.7 (20.1-24.0) | 0.591 |
| Khorana,n(%) |  |  | 0.207 |
| 0 | 157 (26.8) | 124 (21.2) |  |
| 1 | 213 (36.4) | 243 (41.5) |  |
| 2 | 149 (25.5) | 154 (26.3) |  |
| 3 | 54 (9.2) | 51 (8.7) |  |
| 4 | 11 (1.9) | 13 (2.2) |  |
| 5 | 1 (0.2) | 0 (0) |  |
| Khorana ≥ 2, n(%) | 215 (36.8) | 218 (37.3) | 0.856 |
| Tumor stage, n(%) |  |  | 0.609 |
| I | 151 (25.8) | 158 (27.0) |  |
| II | 99 (16.9) | 102 (17.4) |  |
| III | 158 (27.0) | 138 (23.6) |  |
| IV | 177 (30.3) | 187 (32.0) |  |
| Tumor stage ≥ III, n(%) | 335 (57.3) | 325 (55.6) | 0.555 |
| Smoke,n(%) | 83 (14.2) | 78 (13.3) | 0.671 |
| Drink, n(%) | 84 (14.4) | 81 (13.8) | 0.801 |
| History of thrombosis,n(%) | 73 (12.5) | 72 (12.3) | 0.929 |
| Anticoagulation time, median (IQR) | 8 (4-13) | 7 (7-13) | 0.685 |
| Comorbidities, n(%) |  |  |  |
| Hypertension | 175 (29.9) | 153 (26.2) | 0.152 |
| Coronary heart disease | 27 (4.6) | 28 (4.8) | 0.890 |
| Diabetes | 84 (14.4) | 82 (14.0) | 0.867 |
| Congestive heart failure | 10 (1.7) | 8 (1.4) | 0.635 |
| COPD | 15 (2.6) | 12 (2.1) | 0.559 |
| Abnormal renal function | 35 (6.0) | 31 (5.3) | 0.612 |
| Abnormal liver function | 62 (10.6) | 56 (9.6) | 0.560 |
| Atherosclerosis | 55 (9.4) | 52 (8.9) | 0.761 |
| Surgery, n(%)^a^ | 198 (33.8) | 202 (34.5) | 0.805 |
| PICC,n(%) | 232 (39.7) | 228 (39.0) | 0.811 |
| Chemotherapy, n(%) | 248 (42.4) | 258 (44.1) | 0.555 |
| Tumor site, n(%) |  |  |  |
| Lung | 159 (27.2) | 151 (25.8) | 0.596 |
| Cervix | 55 (9.4) | 65 (11.1) | 0.335 |
| Breast | 46 (7.9) | 48 (8.2) | 0.830 |
| Uterus | 22 (3.8) | 31 (5.3) | 0.206 |
| Ovaries | 38 (6.5) | 38 (6.5) | >0.999 |
| Esophagus | 40 (6.8) | 39 (6.7) | 0.906 |
| Rectum | 37 (6.3) | 36 (6.2) | 0.903 |
| Stomach | 41 (7.0) | 38 (6.5) | 0.727 |
| Colon | 29 (5.0) | 28 (4.8) | 0.893 |
| Liver | 24 (4.1) | 20 (3.4) | 0.538 |
| Pancreas | 16 (2.7) | 18 (3.1) | 0.728 |
| Prostate | 12 (2.1) | 10 (1.7) | 0.667 |
| bladder | 9 (1.5) | 11 (1.9) | 0.652 |
| kidney | 7 (1.2) | 7 (1.2) | >0.999 |
| Bile ducts | 5 (0.9) | 4 (0.7) | 0.738 |
| Others ^b^ | 45 (7.7) | 41 (7.0) | 0.653 |
| Chemotherapeutic drug, n(%) |  |  |  |
| Alkylating agents | 123 (21.0) | 133 (22.7) | 0.479 |
| Antimetabolites | 93 (15.9) | 103 (17.6) | 0.434 |
| Antitumor antibiotics | 17 (2.9) | 25 (4.3) | 0.209 |
| Topoisomerase inhibitors | 19 (3.2) | 14 (2.4) | 0.377 |
| Botanical alkaloid | 71 (12.1) | 74 (12.6) | 0.790 |
| Hormone Drugs | 124 (21.2) | 122 (20.9) | 0.886 |
| Molecularly targeted drug | 52 (8.9) | 48 (8.2) | 0.676 |
| Antiplatelet drug/NSAID, n(%) | 112 (19.1) | 110 (18.8) | 0.881 |
| Laboratory indicators, median (IQR) |  |  |  |
| WBC | 6.49 (5.10-8.89) | 6.41 (4.93-8.6) | 0.339 |
| RBC | 4.00 (3.41-4.54) | 3.96 (3.45-4.4) | 0.303 |
| Hb | 119 (101-134) | 118 (104-132) | 0.780 |
| PLT | 224 (171-297) | 227 (171-292) | 0.737 |
| PT | 12.3 (11.4-13.3)) | 12.4 (11.4-13.3) | 0.467 |
| INR | 1.02 (0.94-1.1) | 1.02 (0.94-1.13) | 0.422 |
| APTT | 32.1 (28.6-36.2) | 31.7 (27.3-36.3) | 0.099 |
| D-Dimer | 1.11 (0.43-3.39) | 1.04 (0.44-3.25) | 0.651 |
| Albumin | 37.5 (32.7-41.4) | 38.2 (34.0-41.6) | 0.137 |
| Creatinine | 65 (54-82) | 68 (57-83) | 0.069 |

LMWH: low molecular weight heparin; IQR: interquartile range; BMI: body mass index; COPD: chronic obstructive pulmonary disease; PICC: peripherally inserted central catheter; NSAID: non-steroidal anti-inflammatory drug; WBC: white blood cell count; RBC: red blood cell count; Hb: hemoglobin; PLT: platelet count; PT: prothrombin time; INR: International standardized ratio; APTT: activated partial thromboplastin time

^a^: History of surgery within a month

^b^: Other cancer sites include nasopharynx, fallopian tubes, vagina, ureter, thyroid, tongue, and brain

**Table 3** Baseline characteristics of lung cancer patients after propensity score matching

|  | **All (N=242)** | **LMWH (N=121)** | **Rivaroxaban (N=121)** | **P-value** |
| --- | --- | --- | --- | --- |
| Sex/male, n(%) | 162 (66.9) | 79 (65.3) | 83 (68.6) | 0.585 |
| Age, median (IQR) | 64 (56-70) | 65 (56-71) | 63 (56-70) | 0.336 |
| BMI, median (IQR) | 22.9 (20.2-24.7) | 23.2 (20.2-24.9) | 22.3 (20.2-24.7) | 0.388 |
| Khorana,n(%) |  |  |  | 0.443 |
|  |  |  |  |  |
| 1 | 138 (57.0) | 65 (53.7) | 73 (60.3) |  |
| 2 | 81 (33.5) | 46 (38.0) | 35 (28.9) |  |
| 3 | 17 (7.0) | 8 (6.6) | 9 (7.4) |  |
| 4 | 6(2.5) | 2 (1.7) | 4 (3.3) |  |
|  |  |  |  |  |
| Khorana ≥ 2, n(%) | 104 (42.9) | 56 (46.3) | 48 (39.7) | 0.299 |
| Tumor stage, n(%) |  |  |  | 0.653 |
| 1 | 55 (22.7) | 29 (24.0) | 26 (21.5) |  |
| 2 | 26 (10.7) | 11 (9.1) | 15 (12.4) |  |
| 3 | 47 (19.4) | 21 (17.4) | 26 (21.5) |  |
| 4 | 114 (47.2) | 60 (49.6) | 54 (44.6) |  |
| Tumor stage ≥ 3, n(%) | 161 (66.5) | 81 (66.9) | 80 (66.1) | 0.892 |
| Smoke,n(%) | 56 (23.1) | 31 (25.6) | 25 (20.7) | 0.360 |
| Drink, n(%) | 56 (23.1) | 30 (24.8) | 26 (21.5) | 0.542 |
| History of thrombosis,n(%) | 34 (14.0) | 19 (15.7) | 15 (12.4) | 0.459 |
| Anticoagulation time, median (IQR) | 7 (5-10) | 7 (4-10) | 7 (5-10) | 0.435 |
| Comorbidities, n(%) |  |  |  |  |
| Hypertension | 70 (28.9) | 34 (28.1) | 36 (29.8) | 0.777 |
| Coronary heart disease | 12 (4.9) | 7 (5.8) | 5 (4.1) | 0.554 |
| Diabetes | 19 (7.8) | 5 (4.1) | 14 (11.6) | 0.053 |
| Congestive heart failure | 2 (0.8) | 1 (0.8) | 1 (0.8) | >0.999 |
| COPD | 14 (5.7) | 7 (5.8) | 7 (5.8) | >0.999 |
| Abnormal renal function | 10 (4.1) | 5 (4.1) | 5 (4.1) | >0.999 |
| Abnormal liver function | 22 (9.0) | 8 (6.6) | 14 (11.6) | 0.180 |
| Atherosclerosis | 26 (10.7) | 10 (8.3) | 16 (13.2) | 0.213 |
| Surgery, n(%)^a^ | 53 (21.9) | 27 (22.3) | 26 (21.5) | 0.876 |
| PICC,n(%) | 82 (33.8) | 38 (31.4) | 44 (36.4) | 0.415 |
| Chemotherapy, n(%) | 108 (44.6) | 56 (46.3) | 52 (43.0) | 0.605 |
| Chemotherapeutic drug, n(%) |  |  |  |  |
| Alkylating agents | 57 (23.5) | 28 (23.1) | 29 (24.0) | 0.880 |
| Antimetabolites | 39 (16.1) | 19 (15.7) | 20 (16.5) | 0.861 |
| Antitumor antibiotics | 4 (1.6) | 2 (1.7) | 2 (1.7) | >0.999 |
| Topoisomerase inhibitors | 12 (4.9) | 7 (5.8) | 5 (4.1) | 0.554 |
| Botanical alkaloid | 27 (11.1) | 12 (9.9) | 15 (12.4) | 0.540 |
| Hormone Drugs | 63 (26.0) | 28 (23.1) | 35 (28.9) | 0.305 |
| Molecularly targeted drug | 28 (11.5) | 13 (10.7) | 15 (12.4) | 0.688 |
| Antiplatelet drug/NSAID, n(%) | 52 (21.4) | 26 (21.5) | 26 (21.5) | >0.999 |
| Laboratory indicators, median (IQR) |  |  |  |  |
| WBC | 7.04 (5.34-9.87) | 6.94 (5.30-10.01) | 7.06 (5.54-9.74) | 0.866 |
| RBC | 4.09 (3.45-4.47) | 4.09 (3.64-4.46) | 3.97 (3.42-4.52) | 0.970 |
| Hb | 119 (103-132) | 121 (103-132) | 118 (103-133) | 0.840 |
| PLT | 232 (178-302) | 219 (173-295) | 248 (185-305) | 0.147 |
| PT | 12.4 (11.3-13.5) | 12.5 (11.3-13.6) | 12.4 (11.4-13.3) | 0.968 |
| INR | 1.04 (0.96-1.14) | 1.04 (0.97-1.15) | 1.03 (0.94-1.10) | 0.858 |
| APTT | 32.1 (29.1-36.4) | 31.7 (29.1-35.5) | 32.8 (29.2-37.0) | 0.333 |
| D-Dimer | 15.7 (14.5-17.8) | 15.7 (14.5-17.1) | 15.7 (14.4-17.0) | 0.969 |
| Albumin | 36.5 (32.3-40.4) | 36.2 (31.3-40.4) | 36.8 (33.0-40.0) | 0.389 |
| Creatinine | 68.0 (58.0-84.0) | 67.0 (58.0-80.0) | 69.0 (60.0-87.0) | 0.341 |

LMWH: low molecular weight heparin; IQR: interquartile range; BMI: body mass index; COPD: chronic obstructive pulmonary disease; PICC: peripherally inserted central catheter; NSAID: non-steroidal anti-inflammatory drug; WBC: white blood cell count; RBC: red blood cell count; Hb: hemoglobin; PLT: platelet count; PT: prothrombin time; INR: International standardized ratio; APTT: activated partial thromboplastin time

^a^: History of surgery within a month

**Table 4** Clinical Outcomes of Rivaroxaban and LMWH in lung cancer patients after propensity score matching

| Outcomes | LMWH  (N=121) | Rivaroxaban (N=121) | OR (95% CI) | P-value |
| --- | --- | --- | --- | --- |
| Thrombosis, n(%) | 6 (5.0) | 7 (5.8) | 1.177 (0.384-3.610) | 0.766 |
| VTE, n(%) | 1 (0.8) | 5 (4.1) | 5.172 (0.595-44.947) | 0.098 |
| Other thrombosis, n(%) | 5 (4.1) | 2 (1.7) | 0.390 (0.074-2.050) | 0.250 |
| All bleeding, n(%) | 4 (3.3) | 13 (10.7) | 3.521 (1.114-11.128) | 0.024 |
| Major bleeding, n(%) | 3 (2.5) | 6 (5.0) | 2.052 (0.501-8.401) | 0.308 |
| Non-major bleeding, n(%) | 1 (0.8) | 7 (5.8) | 7.368 (0.893-60.832) | 0.066 |
| All-caused deaths, n(%) | 10 (8.3) | 11 (9.1) | 1.110 (0.453-2.719) | 0.819 |

LMWH: low molecular weight heparin; 95% CI: confidence interval; OR: odds ratio; VTE: venous thromboembolism

**Table 5** Baseline characteristics of tumor surgery patients after propensity score matching

|  | LMWH  (N=168) | Rivaroxaban (N=168) | P-value |
| --- | --- | --- | --- |
| Sex/male, n(%) | 62 (36.9) | 68 (40.5) | 0.502 |
| Age, median (IQR) | 61 (51-67) | 60 (50-67) | 0.917 |
| BMI, median (IQR) | 22.4 (20.5-24.7) | 22.9 (20.3-25.2) | 0.638 |
| Khorana,n(%) |  |  | 0.002 |
| 0 | 60 (35.7) | 29 (17.3) |  |
| 1 | 52 (31.0) | 82 (48.8) |  |
| 2 | 52 (31.0) | 43 (25.6) |  |
| 3 | 12 (7.1) | 13 (7.7) |  |
| 4 | 1 (0.6) | 1 (0.6) |  |
| Khorana ≥ 2, n(%) | 56 (33.3) | 57 (33.9) | 0.908 |
| Tumor stage, n(%) |  |  | 0.919 |
| I | 63 (37.5) | 62 (36.9) |  |
| II | 34 (20.2) | 35 (20.8) |  |
| III | 46 (27.4) | 42 (25.0) |  |
| IV | 25 (14.9) | 29 (17.3) |  |
| Tumor stage ≥ III, n(%) | 71 (42.3) | 71 (42.3) | >0.999 |
| Smoke,n(%) | 9 (5.4) | 17 (10.1) | 0.102 |
| Drink, n(%) | 17 (10.1) | 22 (13.1) | 0.394 |
| History of thrombosis,n(%) | 20 (11.9) | 20 (11.9) | >0.999 |
| Anticoagulation time, median (IQR) | 8 (4-14) | 7 (7-11) | 0.934 |
| Comorbidities, n(%) |  |  |  |
| Hypertension | 36 (21.4) | 39 (23.2) | 0.694 |
| Coronary heart disease | 3 (1.8) | 5 (3.0) | 0.474 |
| Diabetes | 21 (12.5) | 19 (11.3) | 0.736 |
| Congestive heart failure | 2 (1.2) | 0 (0) | 0.499 |
| COPD | 1 (0.6) | 2 (1.2) | >0.999 |
| Abnormal renal function | 4 (2.4) | 5 (3.0) | >0.999 |
| Abnormal liver function | 18 (10.7) | 22 (13.1) | 0.500 |
| Atherosclerosis | 15 (8.9) | 14 (8.3) | 0.846 |
| PICC,n(%) | 88 (52.4) | 98 (58.3) | 0.272 |
| Tumor site, n(%) |  |  | 1.000 |
| Lung | 29 (17.3) | 35 (20.8) |  |
| Cervix | 32 (19.0) | 28 (16.7) |  |
| Breast | 15 (8.9) | 17 (10.1) |  |
| Uterus | 16 (9.5) | 14 (8.3) |  |
| Ovaries | 10 (6.0) | 12 (7.1) |  |
| Esophagus | 8 (4.8) | 7 (4.2) |  |
| Rectum | 10 (6.0) | 10 (6.0) |  |
| Stomach | 7 (4.2) | 8 (4.8) |  |
| Colon | 3 (1.8) | 4 (2.4) |  |
| Liver | 11 (6.5) | 8 (4.8) |  |
| Pancreas | 5 (3.0) | 5 (3.0) |  |
| Prostate | 3 (1.8) | 2 (1.2) |  |
| bladder | 5 (3.0) | 5 (3.0) |  |
| kidney | 3 (1.8) | 2 (1.2) |  |
| Others ^a^ | 11 (6.5) | 11 (6.5) |  |
| Chemotherapy, n(%) | 50 (29.8) | 54 (32.1) | 0.637 |
| Chemotherapeutic drug, n(%) |  |  |  |
| Alkylating agents | 31 (18.5) | 34 (20.2) | 0.679 |
| Antimetabolites | 18 (10.7) | 17 (10.1) | 0.858 |
| Antitumor antibiotics | 8 (4.8) | 9 (5.4) | 0.803 |
| Topoisomerase inhibitors | 1 (0.6) | 1 (0.6) | >0.999 |
| Botanical alkaloid | 8 (4.8) | 13 (7.7) | 0.260 |
| Hormone Drugs | 38 (22.6) | 38 (22.6) | >0.999 |
| Molecularly targeted drug | 5 (3.0) | 4 (2.4) | >0.999 |
| Antiplatelet drug/NSAID, n(%) | 27 (16.1) | 29 (17.3) | 0.770 |
| Laboratory indicators, median (IQR) |  |  |  |
| WBC | 6.07 (4.63-7.81) | 6.18 (4.77-8.20) | 0.687 |
| RBC | 4.13 (3.65-4.58) | 4.19 (3.67-4.48) | 0.915 |
| Hb | 122 (104-135) | 122 (104-135) | 0.565 |
| PLT | 232 (182-295) | 230 (180-294) | 0.704 |
| PT | 12.0 (11.3-13.0) | 12.2 (11.4-13.0) | 0.431 |
| INR | 0.98 (0.90-1.04) | 0.97 (0.91-1.04) | 0.621 |
| APTT | 31.0 (27.4-34.4) | 32.9 (27.0-36.7) | 0.899 |
| D-Dimer | 0.72 (0.31-1.78) | 0.69 (0.38-2.25) | 0.560 |
| Albumin | 40.1 (35.7-43.3) | 39.5 (35.9-42.6) | 0.793 |
| Creatinine | 64 (54-77) | 63 (56-73) | 0.402 |

LMWH: low molecular weight heparin; IQR: inter quartile range; BMI: body mass index; COPD: chronic obstructive pulmonary disease; PICC: peripherally inserted central catheter; NSAID: non-steroidal anti-inflammatory drug; WBC: white blood cell count; RBC: red blood cell count; Hb: hemoglobin; PLT: platelet count; PT: prothrombin time; INR: International standardized ratio; APTT: activated partial thromboplastin time

^a^: Other cancer sites include nasopharynx, fallopian tubes, vagina, ureter, thyroid, tongue, and brain

**Table 6** Clinical Outcomes of Rivaroxaban and LMWH in tumor surgery patients after propensity score matching

| **Outcomes** | **LMWH (N=168)** | **Rivaroxaban (N=168)** | **P-value** |
| --- | --- | --- | --- |
| Thrombosis, n(%) | 7 (4.2) | 5 (3.0) | 0.557 |
| VTE, n(%) | 5 (3.0) | 1 (0.6) | 0.215 |
| Other thrombosis, n(%) | 2 (1.2) | 4 (2.4) | 0.685 |
| All bleeding, n(%) | 9 (5.4) | 5 (3.0) | 0.275 |
| Major bleeding, n(%) | 4 (2.4) | 0 (0) | 0.123 |
| Non-major bleeding, n(%) | 5 (3.0) | 5 (3.0) | >0.999 |
| All-caused deaths, n(%) | 0 (0) | 6 (3.6) | 0.030 |

LMWH: low molecular weight heparin; VTE: venous thromboembolism

**Table 7** Baseline characteristics of chemotherapy patients after propensity score matching

|  | **LMWH**  **(N=216)** | **Rivaroxaban**  **(N=216)** | **P-value** |
| --- | --- | --- | --- |
| Sex/male, n(%) | 107 (49.5) | 106 (49.1) | 0.923 |
| Age, median (IQR) | 60 (52-68) | 60 (49-66) | 0.564 |
| BMI, median (IQR) | 22.2 (20.0-24.7) | 22.8 (20.2-24.7) | 0.659 |
| Khorana,n(%) |  |  | 0.816 |
| 0 | 49 (22.7) | 44 (20.4) |  |
| 1 | 77 (35.6) | 82 (38.0) |  |
| 2 | 69 (31.9) | 74 (34.3) |  |
| 3 | 17 (7.9) | 14 (6.5) |  |
| 4 | 4 (1.9) | 2 (0.9) |  |
| Khorana ≥ 2, n(%) | 90 (41.7) | 90 (41.7) | >0.999 |
| Tumor stage, n(%) |  |  | 0.985 |
| I | 37 (17.1) | 38 (17.6) |  |
| II | 36 (16.7) | 35 (16.2) |  |
| III | 65 (30.1) | 62 (28.7) |  |
| IV | 78 (36.1) | 81 (37.5) |  |
| Tumor stage ≥ III, n(%) | 143 (66.2) | 143 (66.2) | >0.999 |
| Smoke,n(%) | 32 (14.8) | 31 (14.4) | 0.892 |
| Drink, n(%) | 34 (15.7) | 26 (12.0) | 0.266 |
| History of thrombosis,n(%) | 33 (15.3) | 31 (14.4) | 0.786 |
| Anticoagulation time, median (IQR) | 8 (3-13) | 7 (4-14) | 0.935 |
| Comorbidities, n(%) |  |  |  |
| Hypertension | 50 (23.1) | 49 (22.7) | 0.909 |
| Coronary heart disease | 6 (2.8) | 5 (2.3) | 0.760 |
| Diabetes | 25 (11.6) | 21 (9.7) | 0.533 |
| Congestive heart failure | 1 (0.5) | 2 (0.9) | >0.999 |
| COPD | 3 (1.4) | 2 (0.9) | >0.999 |
| Abnormal renal function | 7 (3.2) | 11 (5.1) | 0.336 |
| Abnormal liver function | 21 (9.7) | 26 (12.0) | 0.440 |
| Atherosclerosis | 17 (7.9) | 16 (7.4) | 0.856 |
| Surgery, n(%)^a^ | 59 (27.3) | 58 (26.9) | 0.914 |
| PICC,n(%) | 103 (47.7) | 95 (44.0) | 0.440 |
| Tumor site, n(%) |  |  | 0.916 |
| Lung | 61 (28.2) | 52 (24.1) |  |
| Cervix | 16 (7.4) | 18 (8.3) |  |
| Breast | 23 (10.6) | 28 (13.0) |  |
| Uterus | 6 (2.8) | 6 (2.8) |  |
| Ovaries | 13 (6.0) | 18 (8.3) |  |
| Esophagus | 18 (8.3) | 14 (6.5) |  |
| Rectum | 12 (5.6) | 16 (7.4) |  |
| Stomach | 14 (6.5) | 13 (6.0) |  |
| Colon | 19 (8.8) | 10 (4.6) |  |
| Liver | 2 (0.9) | 4 (1.9) |  |
| Pancreas | 7 (3.2) | 7 (3.2) |  |
| Prostate | 2 (0.9) | 3 (1.4) |  |
| bladder | 3 (1.4) | 5 (2.3) |  |
| kidney | 2 (0.9) | 1 (0.5) |  |
| Bile ducts | 1 (0.5) | 2 (0.9) |  |
| Others ^b^ | 17 (7.9) | 19 (8.8) |  |
| Chemotherapeutic drug, n(%) |  |  |  |
| Alkylating agents | 109 (50.5) | 111 (51.4) | 0.847 |
| Antimetabolites | 86 (39.8) | 72 (33.3) | 0.162 |
| Antitumor antibiotics | 14 (6.5) | 16 (7.4) | 0.705 |
| Topoisomerase inhibitors | 17 (7.9) | 15 (6.9) | 0.713 |
| Botanical alkaloid | 59 (27.3) | 63 (29.2) | 0.669 |
| Hormone Drugs | 73 (33.8) | 63 (29.2) | 0.300 |
| Molecularly targeted drug | 48 (22.2) | 49 (22.7) | 0.908 |
| Antiplatelet drug/NSAID, n(%) | 36 (16.7) | 34 (15.7) | 0.974 |
| Laboratory indicators, median (IQR) |  |  |  |
| WBC | 6.00 (4.82-8.22) | 6.07 (4.38-8.10) | 0.248 |
| RBC | 3.80 (3.30-4.38) | 3.92 (3.4-4.41) | 0.120 |
| Hb | 112 (97-130) | 116 (102-132) | 0.144 |
| PLT | 227 (171-298) | 230 (168-293) | 0.927 |
| PT | 12.3 (11.3-13.3) | 11.9 (10.8-13.1) | 0.481 |
| INR | 1.01 (0.93-1.10) | 0.98 (0.90-1.06) | 0.549 |
| APTT | 31.8 (27.8 -37.2) | 29.8 (25.7034.8) | 0.415 |
| D-Dimer | 1.37 (0.53-3.65) | 1.48 (0.56-3.69) | 0.993 |
| Albumin | 37.9 (33.2-41.5) | 38.2 (34.4-41.2) | 0.565 |
| Creatinine | 64 (55-78) | 66 (57-83) | 0.260 |

LMWH: low molecular weight heparin; IQR: interquartile range; BMI: body mass index; COPD: chronic obstructive pulmonary disease; PICC: peripherally inserted central catheter; NSAID: non-steroidal anti-inflammatory drug; WBC: white blood cell count; RBC: red blood cell count; Hb: hemoglobin; PLT: platelet count; PT: prothrombin time; INR: International standardized ratio; APTT: activated partial thromboplastin time

^a^: History of surgery within a month

^b^: Other cancer sites include nasopharynx, fallopian tubes, vagina, ureter, thyroid, tongue, and brain

**Table 8** Clinical Outcomes of Rivaroxaban and LMWH in chemotherapy patients after propensity score matching

| **Outcomes** | **LMWH (N=216)** | **Rivaroxaban (N=216)** | **OR (95% CI)** | **P-value** |
| --- | --- | --- | --- | --- |
| Thrombosis, n(%) | 19 (8.8) | 8 (3.7) | 0.399 (0.171-0.932) | 0.029 |
| VTE, n(%) | 7 (3.2) | 5 (2.3) | 0.807 (0.221-2.265) | 0.558 |
| Other thrombosis, n(%) | 12 (5.6) | 3 (1.4) | 0.239 (0.067-0.861) | 0.018 |
| All bleeding, n(%) | 17 (7.9) | 16 (7.4) | 0.936 (0.460-1.905) | 0.856 |
| Major bleeding, n(%) | 2 (0.9) | 6 (2.8) | 3.057 (0.610-15.319) | 0.153 |
| Non-major bleeding, n(%) | 15 (6.9) | 10 (4.6) | 0.650 (0.286-1.482) | 0.303 |
| All-caused deaths, n(%) | 8 (3.7) | 9 (4.2) | 1.130 (0.428-2.987) | 0.805 |

LMWH: low molecular weight heparin; 95% CI: confidence interval; OR: odds ratio; VTE: venous thromboembolism

**Table 9** Chemotherapy times and thrombotic events in patients treated with chemotherapy in the rivaroxaban group

| **Chemotherapy times (n=304)** | **Thrombosis (n=34) / n(%)** | **P value** |
| --- | --- | --- |
| 1-2 (n=133) | 11 (8.3) | Reference |
| 3-4 (n=54) | 10 (18.5) | 0.044 |
| 5-6 (n=47) | 5 (10.6) | 0.624 |
| 7-8 (n=31) | 2 (6.5) | 0.735 |
| ≥ 9 (n=39) | 6 (15.4) | 0.190 |

**Table 10** Chemotherapy times and thrombotic events in patients treated with chemotherapy in the LMWH group

| **Chemotherapy times (n=431)** | **Thrombosis (n=29) / n(%)** | **P value** |
| --- | --- | --- |
| 1-2 (n=214) | 21 (9.8) | Reference |
| 3-4 (n=99) | 6 (6.1) | 0.271 |
| 5-6 (n=63) | 2 (3.2) | 0.093 |
| 7-8 (n=35) | 0 (0) | 0.053 |
| ≥ 9 (n=20) | 0 (0) | 0.142 |

# Supplementary Figures


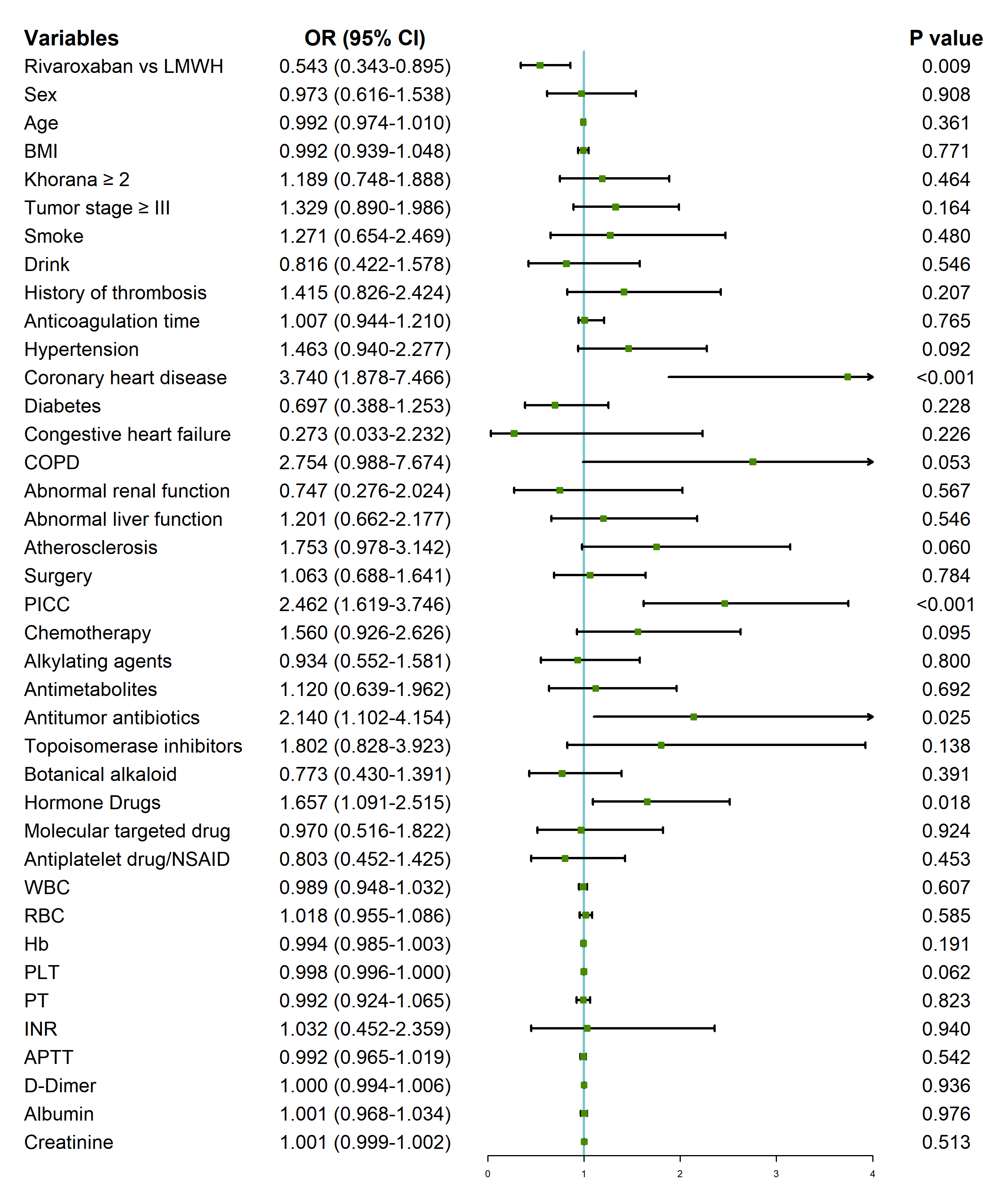


**Supplementary Figures 1** Association of anticoagulants and potential risk factors with thrombosis in cancer patients

95% CI: confidence interval; OR: odds ratio; LMWH: low molecular weight heparin; BMI: body mass index; COPD: chronic obstructive pulmonary disease; PICC: peripherally inserted central catheter; NSAID: non-steroidal anti-inflammatory drug; WBC: white blood cell count; RBC: red blood cell count; Hb: hemoglobin; PLT: platelet count; PT: prothrombin time; INR: International standardized ratio; APTT: activated partial thromboplastin time


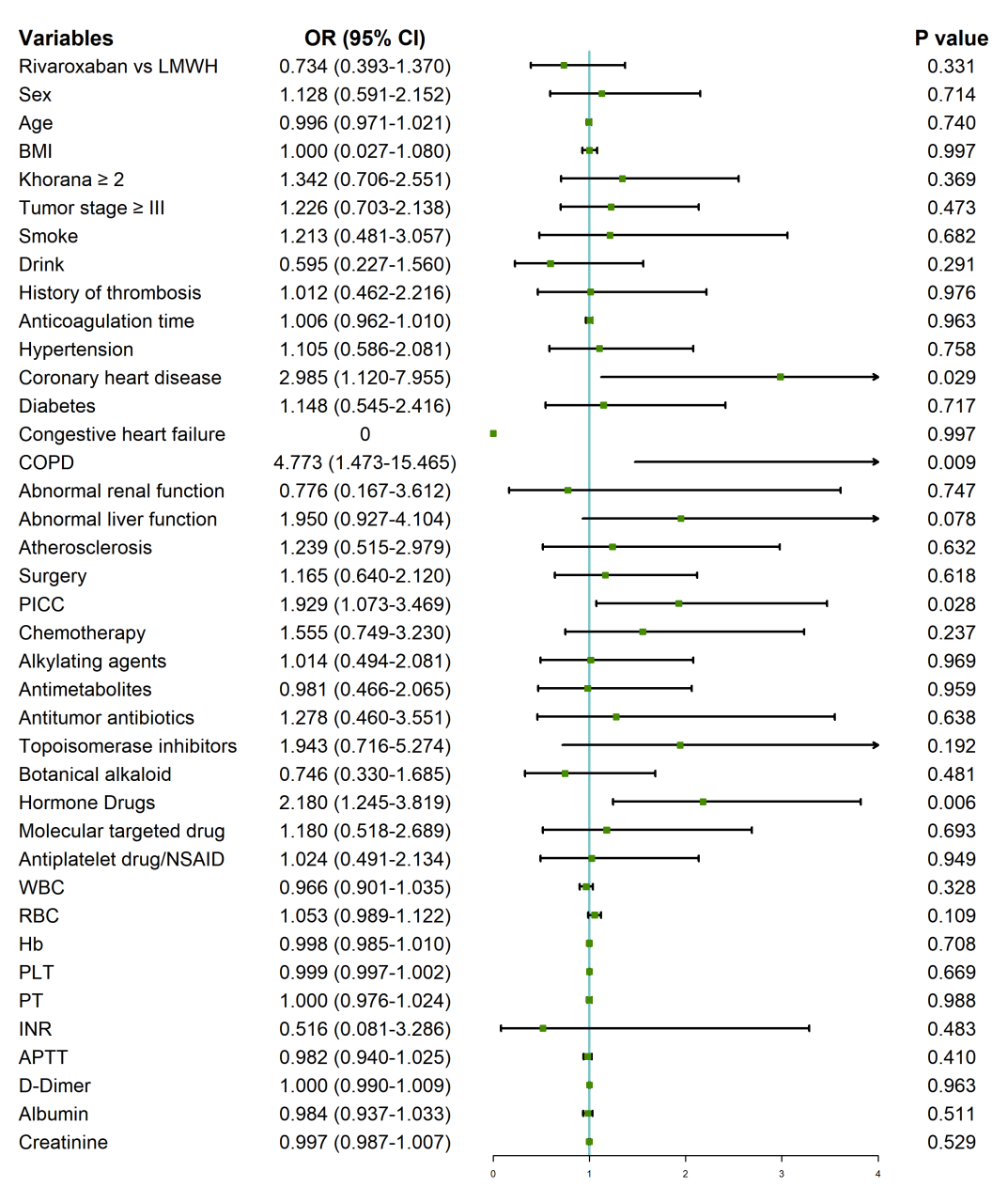


**Supplementary Figures 2** Association of anticoagulants and potential risk factors with VTE in cancer patients

95% CI: confidence interval; OR: odds ratio; LMWH: low molecular weight heparin; BMI: body mass index; COPD: chronic obstructive pulmonary disease; PICC: peripherally inserted central catheter; NSAID: non-steroidal anti-inflammatory drug; WBC: white blood cell count; RBC: red blood cell count; Hb: hemoglobin; PLT: platelet count; PT: prothrombin time; INR: International standardized ratio; APTT: activated partial thromboplastin time


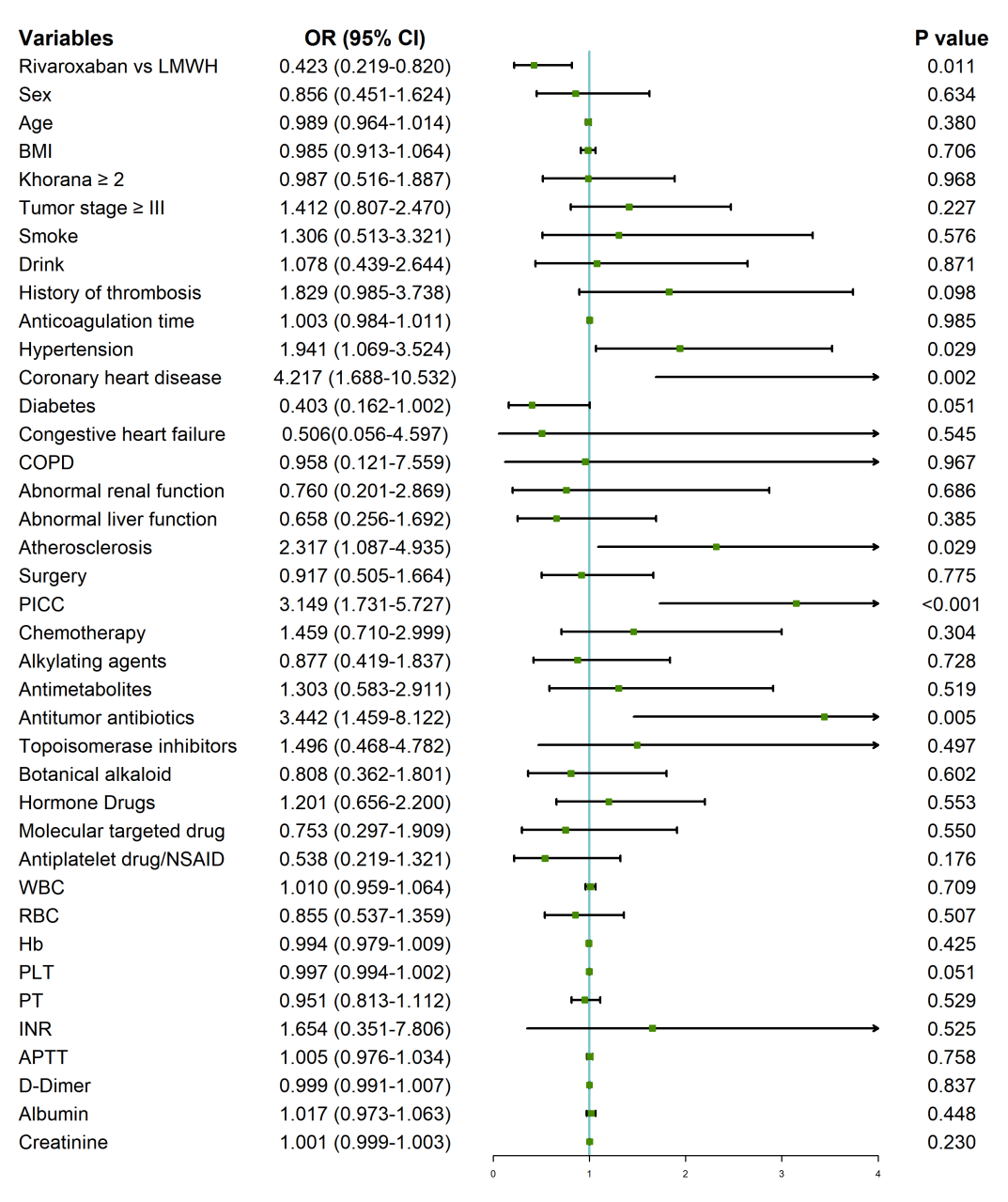


**Supplementary Figures 3** Association of anticoagulants and potential risk factors with other thrombosis in cancer patients

95% CI: confidence interval; OR: odds ratio; LMWH: low molecular weight heparin; BMI: body mass index; COPD: chronic obstructive pulmonary disease; PICC: peripherally inserted central catheter; NSAID: non-steroidal anti-inflammatory drug; WBC: white blood cell count; RBC: red blood cell count; Hb: hemoglobin; PLT: platelet count; PT: prothrombin time; INR: International standardized ratio; APTT: activated partial thromboplastin time


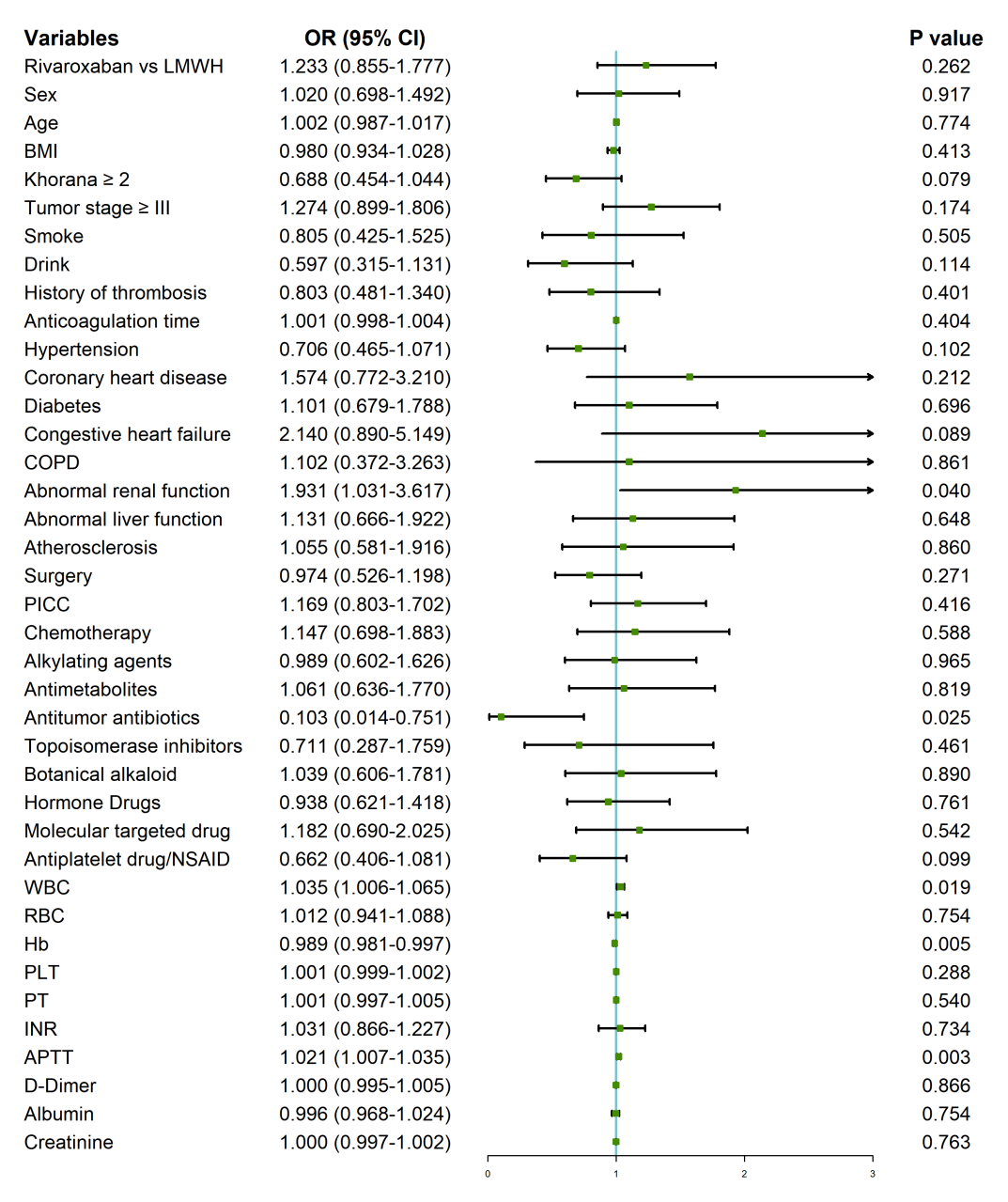


**Supplementary Figures 4** Association of anticoagulants and potential risk factors with all bleeding in cancer patients

95% CI: confidence interval; OR: odds ratio; LMWH: low molecular weight heparin; BMI: body mass index; COPD: chronic obstructive pulmonary disease; PICC: peripherally inserted central catheter; NSAID: non-steroidal anti-inflammatory drug; WBC: white blood cell count; RBC: red blood cell count; Hb: hemoglobin; PLT: platelet count; PT: prothrombin time; INR: International standardized ratio; APTT: activated partial thromboplastin time


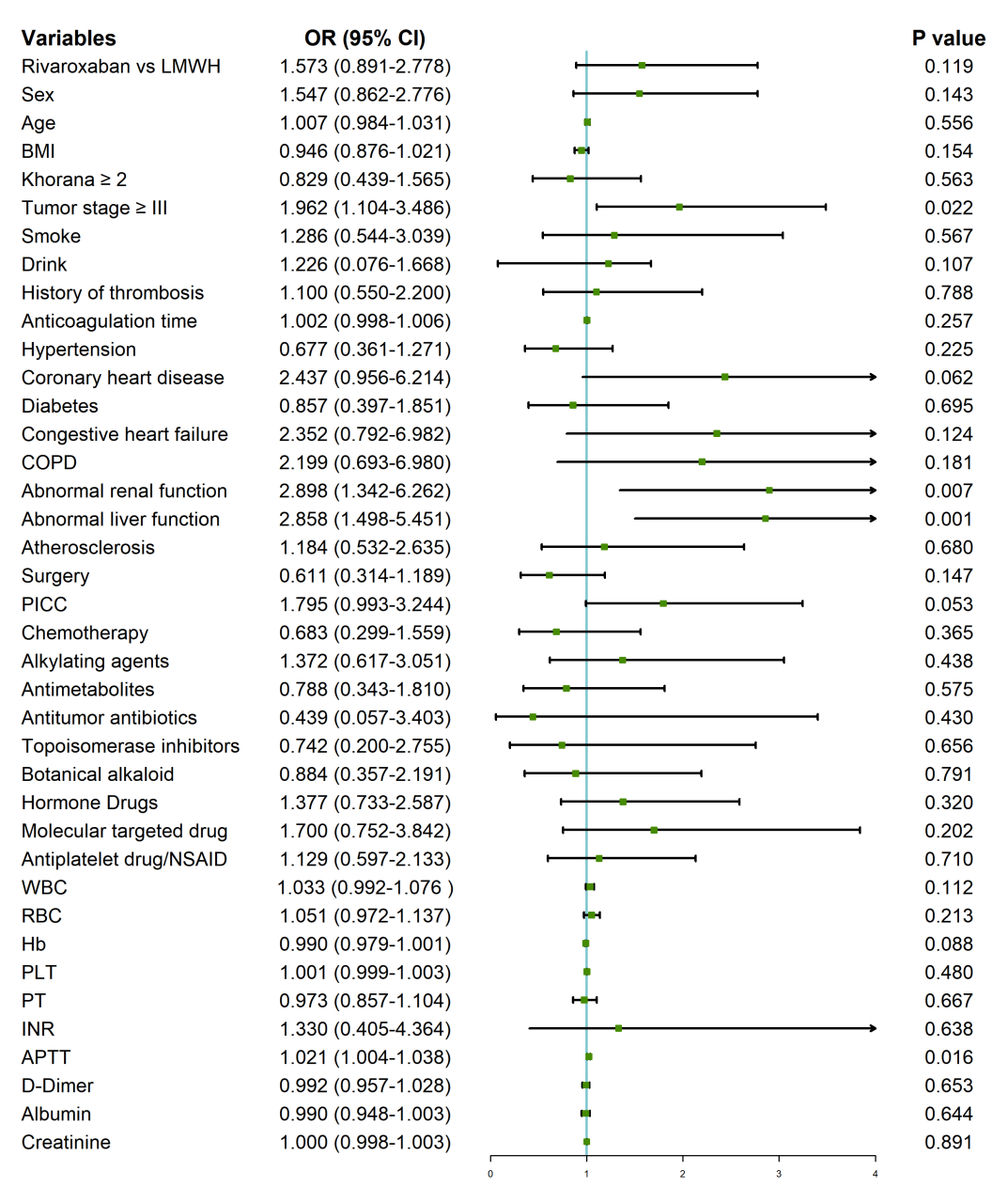


**Supplementary Figures 5** Association of anticoagulants and potential risk factors with major bleeding in cancer patients

95% CI: confidence interval; OR: odds ratio; LMWH: low molecular weight heparin; BMI: body mass index; COPD: chronic obstructive pulmonary disease; PICC: peripherally inserted central catheter; NSAID: non-steroidal anti-inflammatory drug; WBC: white blood cell count; RBC: red blood cell count; Hb: hemoglobin; PLT: platelet count; PT: prothrombin time; INR: International standardized ratio; APTT: activated partial thromboplastin time


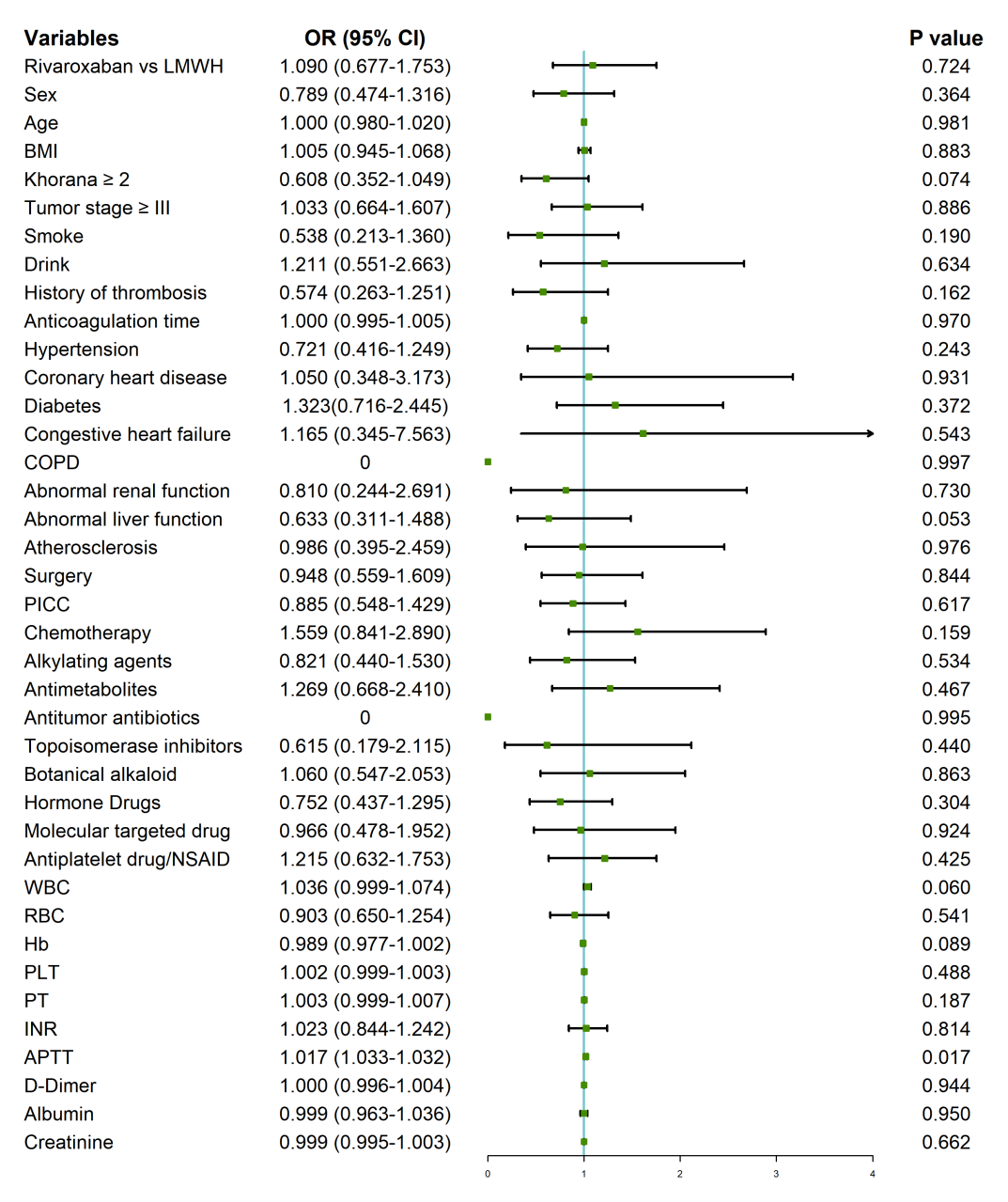


**Supplementary Figures 6** Association of anticoagulants and potential risk factors with minor bleeding in cancer patients

95% CI: confidence interval; OR: odds ratio; LMWH: low molecular weight heparin; BMI: body mass index; COPD: chronic obstructive pulmonary disease; PICC: peripherally inserted central catheter; NSAID: non-steroidal anti-inflammatory drug; WBC: white blood cell count; RBC: red blood cell count; Hb: hemoglobin; PLT: platelet count; PT: prothrombin time; INR: International standardized ratio; APTT: activated partial thromboplastin time


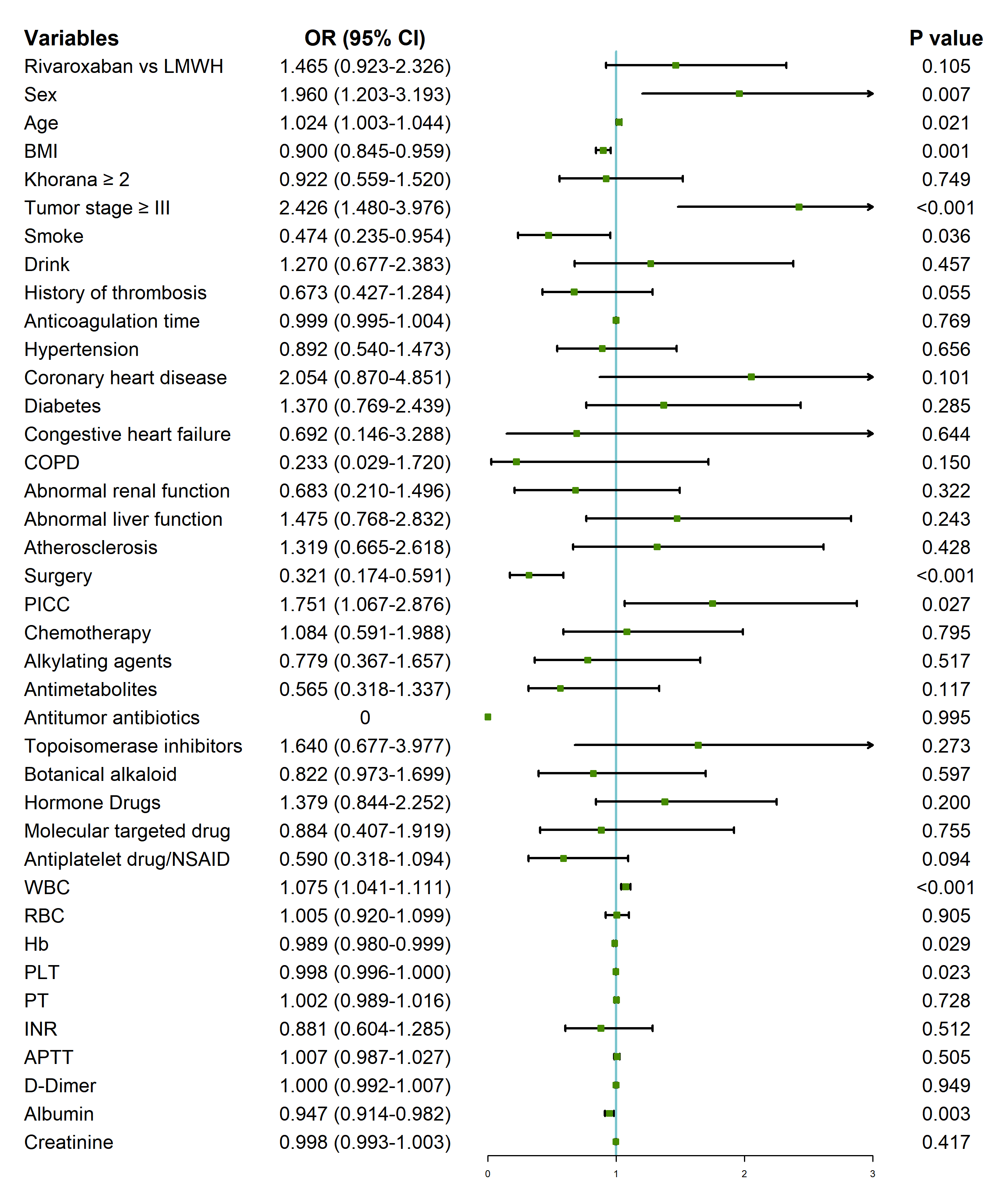


**Supplementary Figures 7** Association of anticoagulants and potential risk factors with all-cause deaths in cancer patients

95% CI: confidence interval; OR: odds ratio; LMWH: low molecular weight heparin; IBMI: body mass index; COPD: chronic obstructive pulmonary disease; PICC: peripherally inserted central catheter; NSAID: non-steroidal anti-inflammatory drug; WBC: white blood cell count; RBC: red blood cell count; Hb: hemoglobin; PLT: platelet count; PT: prothrombin time; INR: International standardized ratio; APTT: activated partial thromboplastin time
